# Ribosome Biogenesis as a Putative Bottleneck to Skeletal Muscle Hypertrophy: Mechanisms, Human Evidence, and Practical Modulators

**DOI:** 10.3390/cells15111041

**Published:** 2026-06-05

**Authors:** Mario Muñoz López, José Francisco López-Gil, Xabier Ramírez de la piscina Viúdez, Eneko Baz-Valle, José Francisco Tornero Aguilera

**Affiliations:** 1 Department of Sport Sciences, Faculty of Sport and Health Sciences, Fit Generation Research Institute, AD500 Andorra la Vella, Andorra; mario.mlopez@fitgeneration.es (M.M.L.); xabier.ramirezpiscina@gmail.com (X.R.d.l.p.V.); enekowushu@gmail.com (E.B.-V.); 2 School of Medicine, Universidad Espíritu Santo, Samborondón 092301, Ecuador; josefranciscolopezgil@gmail.com; 3 Faculty of Health Sciences, Universidad Autónoma de Chile, Temuco 4780000, Chile

**Keywords:** ribosome biogenesis, skeletal muscle hypertrophy, translational capacity, RNA Polymerase I, nucleolus, mechanotransduction, resistance training

## Abstract

**Highlights:**

**What are the main findings?**

- Translational capacity, not signaling efficiency, is the true hypertrophic bottleneck: Sustained skeletal muscle hypertrophy requires the physical expansion of nucleolar factories driven by RNA Polymerase I transcription and ribosome biogenesis, rather than transient mTORC1 activation; high responders to resistance training are distinguished by their capacity to accumulate ribosomal material across training blocks.
- Methodological artifacts systematically underestimate ribosomal adaptation: The pervasive moving denominator paradox—normalizing total RNA to wet tissue mass in biopsies from hypertrophied muscle—obscures absolute ribosomal expansion, meaning that a substantial portion of the human literature may have underestimated the true magnitude of nucleolar adaptation to resistance training.

**What are the implications of the main findings?**

- Training design should prioritize recoverable mechanical volume and energy availability over time: Nucleolar expansion is an inherently slow structural process; practitioners should expect meaningful ribosomal accretion to unfold across weeks to months of consistent loading, not within isolated sessions. Sufficient weekly volume (≥10 hard sets per muscle group), separation of concurrent endurance training by 6–24 h, and adequate energy availability are the environmental conditions required to sustain this gradual factory expansion across a training block.
- Muscle memory has a structural and epigenetic basis that should inform long-term programming: Myonuclear addition and epigenetic hypomethylation of rDNA promoters establish a durable architectural foundation that accelerates retraining responses, supporting periodization strategies that preserve training history rather than treating each block in isolation.

**Abstract:**

Background: Skeletal muscle hypertrophy has traditionally been attributed to transient spikes in translational efficiency governed by the mTORC1 signaling cascade. However, contemporary molecular evidence reveals that sustained macroscopic growth is strongly associated with the physical expansion of the translational machinery itself. The activation of RNA Polymerase I and the subsequent synthesis of new ribosomes represent a critical biological correlate for long-term protein accretion. Objective: This comprehensive review critically examines ribosome biogenesis as the primary structural bottleneck shaping human skeletal muscle adaptation, differentiating acute signaling efficiency from chronic translational capacity. Synthesis: We dissect the molecular orchestration of nucleolar expansion and critically address the pervasive methodological pitfalls plaguing the current literature. Specifically, we highlight the moving denominator paradox, demonstrating how flawed bulk RNA normalization strategies systematically underestimate true ribosomal accretion in actively growing tissue. By synthesizing in vivo human evidence, we delineate how age, concurrent training, and training volume modulate this structural capacity. We further establish the high-responder phenotype as a function of successful nucleolar adaptation. Finally, we explore advanced molecular frontiers, including epigenetic chromatin remodeling, ribosomal heterogeneity as an emerging frontier, non-coding RNA regulation, and nuclear mechanotransduction via the YAP/TAZ axis. Conclusions: Acute anabolic signaling is merely permissive. Permanent hypertrophic adaptation fundamentally relies on overcoming the translational capacity bottleneck. Shifting the scientific and applied focus toward the architectural expansion of the nucleolus will fundamentally redefine practical hypertrophy programming and clinical interventions for sarcopenia.

## 1. Introduction

Skeletal muscle constitutes the primary amino acid reservoir of the human body, accounting for approximately 40% of body mass and operating not only as the fundamental engine for locomotion and force generation but also as an indispensable metabolic sink for glycemic and lipid homeostasis [1,2,3,4]. In high-performance sports, expansion of muscle cross-sectional area (hypertrophy) is a primary determinant of maximal power output and mechanical resilience [5,6]. In stark contrast, from a clinical and epidemiological perspective, preservation of the myofibrillar network serves as a critical biological buffer against metabolic dysfunction [7,8,9]. Progressive muscle atrophy, whether induced by mechanical disuse, aging (sarcopenia), or chronic disease states such as cancer cachexia, drastically increases all-cause morbidity and mortality rates [10,11,12]. At the cellular level, phenotypic plasticity of skeletal muscle, transitioning between hypertrophy and atrophy, is dictated by the long-term kinetic equilibrium between muscle protein synthesis (MPS) and muscle protein breakdown (MPB) [13,14].

Over the past two decades, exercise physiology and biomedicine have investigated this anabolic–catabolic balance predominantly through the paradigm of *translational efficiency* [15,16]. This historical model focuses on the rate at which pre-existing cellular machinery translates messenger RNA (mRNA) into new functional proteins [17]. Consequently, an extraordinarily influential body of literature has been generated detailing how mechanical tension, amino acid availability, and growth factors acutely activate the mechanistic target of rapamycin complex 1 (mTORC1) and its downstream effectors (p70S6K and 4E-BP1) to temporarily accelerate peptide assembly [18,19,20]. However, modern longitudinal research has exposed a profound “kinetic disconnect” within this model: acute spikes in mTORC1 signaling and transient post-exercise elevations in MPS frequently fail to reliably correlate with or predict the magnitude of macroscopic hypertrophy following months of continuous training [21,22,23]. Long-term growth is not a simple mathematical summation of acute fluctuations in enzymatic efficiency [24].

To resolve this predictive limitation, contemporary muscle biology has undergone a paradigm shift toward *translational capacity*, defined as the total physical abundance of functional ribosomes available within the sarcoplasm to decode mRNA [25,26]. Because mature muscle fibers are post-mitotic, multinucleated syncytia that cannot undergo cellular division, the accumulation of contractile mass relies on a massive intracellular infrastructure [27,28]. This structural capacity is governed by ribosome biogenesis, a thermodynamically demanding, orchestrated nucleolar process that coordinates the activity of all three nuclear RNA polymerases [29,30]. The rate-limiting step is the transcription of ribosomal DNA (rDNA) by RNA polymerase I (Pol I) to produce the 45S pre-rRNA precursor, which, following intricate processing guided by small nucleolar RNAs (snoRNAs), assembles with ribosomal proteins and the 5S rRNA into mature subunits [31,32]. Therefore, the size of the ribosomal pool establishes an absolute, structural ceiling on the maximal synthetic rate achievable by the cell [33].

Recent evidence has demonstrated that translational control and ribosome biogenesis are not solely mechanisms of anabolic adaptation but rather operate as bidirectional determinants of muscle plasticity across its entire spectrum [34]. On one hand, the early accumulation of total RNA and mature rRNA species during the initial weeks of mechanical overload obligatorily precedes phenotypic hypertrophy, explaining the well-documented observation that some individuals develop substantially greater muscle hypertrophy than others even when subjected to identical resistance-training volumes [35,36]. In contrast, recent clinical literature reveals that nucleolar failure, impaired ribosome production, and the repression of Pol I transcription causally precede and predict muscle atrophy in models of disuse and myopathies [37,38]. While the classic dogma attributed muscle loss almost exclusively to proteolytic pathways, it is now evident that the collapse of translational capacity is a critical determinant and structurally limiting factor that contributes to tissue wasting [39,40].

An important epistemic caveat applies to the framework that follows. The argument that translational capacity is a critical determinant and structurally limiting factor for skeletal-muscle hypertrophy rests on a body of evidence that is mechanistically coherent and quantitatively reproducible, but that remains, in humans, predominantly correlative. Direct causal experiments—selective perturbation of ribosomal biogenesis at constant mechanical and nutritional input—are currently feasible only in preclinical models. Ribosome biogenesis is therefore best understood as a critical determinant and key correlate of sustained hypertrophy, with the recognition that strict causality in humans awaits intervention studies designed to manipulate translational capacity independently of upstream signalling.

Bridging the gap between macroscopic mechanical loading and nucleolar transcription requires a precise understanding of nuclear mechanotransduction, a critical step that is frequently overlooked in applied sports science [41]. Mechanical tension applied to the sarcolemma is not communicated to the nucleolus via simple diffusion of kinases; rather, it requires direct physical mechanocoupling [42]. The deformation of the cytoskeleton is transmitted directly to the nuclear envelope via the Linker of Nucleoskeleton and Cytoskeleton (LINC) complex. This structural strain alters nuclear architecture, facilitating chromatin remodeling and permitting the nuclear translocation of mechanosensitive transcriptional co-activators like YAP and TAZ. Once in the nucleus, YAP/TAZ obligatorily interact with TEAD transcription factors to strongly drive the expression of c-Myc. Subsequently, c-Myc acts as the direct effector, binding to rDNA promoters to orchestrate Pol I activity and nucleolar expansion [43,44]. Understanding this mechanobiological connection confirms mechanical loading as the primary upstream driver of ribosome biogenesis [45].

This structural dependency acquires critical relevance in the context of human aging, manifesting clinically as “anabolic resistance” [46]. As skeletal muscle ages, the cytoskeletal network and nucleolus experience profound desensitization to mechanical stimuli that would normally trigger ribosome biogenesis. Older adults subjected to identical resistance training protocols as younger cohorts exhibit significantly blunted accumulation of total RNA and attenuated transcription of the 45S pre-rRNA precursor, thereby severely limiting their hypertrophic potential [47]. This senescent inability to expand cellular infrastructure underscores that hypertrophy fails not merely due to a lack of acute signaling, but because of a rigid architectural ceiling that decreases with age, rendering the nucleolus a primary therapeutic target to combat sarcopenia.

Despite the overwhelming physiological importance of this dual process, the translational application of ribosome biogenesis currently suffers from severe methodological biases and dangerous over-extrapolation [48]. In vivo measurement of ribosomal turnover in humans frequently forces researchers to rely on bulk total RNA quantification from percutaneous biopsies [49]. However, the flawed mathematical normalization of these nucleic acids to wet tissue weight, ignoring training-induced sarcoplasmic dilution, has plagued the literature with false negatives [50]. Compounding this issue, the sports nutrition industry has co-opted these concepts, erroneously assuming that specific dietary interventions (e.g., supramaximal protein dosing) directly modulate nucleolar rDNA transcription, when rigorous evidence demonstrates that they operate solely as permissive substrates [51,52].

To systematically address these empirical deficiencies and build upon recent bidirectional models in the field [37], this narrative review pursues comprehensive objectives. First, we critically synthesize the molecular biology of ribosome biogenesis, delineating the strict dichotomy between translational efficiency and capacity. Second, we evaluate longitudinal in vivo evidence positioning nucleolar dynamics as the primary biological bottleneck in both hypertrophy and atrophy. Finally, we rigorously dissect the practical modulators of this response, drawing a strict, evidence-based line between established physiological requirements (e.g., training volume) and speculative, performance-oriented feeding practices. By emphasizing methodological rigor over reductionist applications, this review aims to fundamentally refine how sport scientists, clinicians, and practitioners integrate molecular mechanisms into applied hypertrophy programming.

## 2. Literature Search Strategy and Evidence Framework

To systematically synthesize the evolving conceptual transition from translational efficiency to translational capacity in skeletal muscle plasticity, this article was designed as a comprehensive narrative review. A narrative, integrative approach was explicitly selected over a traditional meta-analysis because of the profound methodological heterogeneity characterizing the field—specifically, the vast discrepancies in RNA extraction protocols, normalization strategies (e.g., wet tissue weight versus DNA content), and biopsy timing across human trials. Attempting to compute a pooled quantitative effect size from such disparate molecular data would obscure crucial biological nuances. Instead, our objective was to rigorously map the mechanistic continuum connecting mechanotransduction, nucleolar dynamics, and macroscopic muscle phenotypes (hypertrophy and atrophy).

A highly structured literature search was conducted across primary scientific databases, including PubMed/MEDLINE, Web of Science Core Collection, Scopus, and Embase. To capture the full historical evolution of muscle protein synthesis research—from the foundational discoveries of the mTOR signaling cascade to the contemporary focus on RNA Polymerase I and nucleolar architecture—the search timeline was deliberately expansive, covering literature published from January 1990 to March 2026. The search strategy utilized complex Boolean logic strings to intersect core conceptual domains. Primary keywords included combinations of (“ribosome biogenesis” OR “translational capacity” OR “nucleolar function” OR “45S pre-rRNA”) AND (“skeletal muscle” OR “myofiber” OR “hypertrophy” OR “atrophy” OR “sarcopenia”) AND (“resistance training” OR “mechanical loading” OR “stretch loading” OR “stretch overload” OR “eccentric training” OR “mechanotransduction” OR “concurrent training”). Furthermore, forward and backward citation tracking of seminal papers and highly cited reviews was conducted to ensure absolute literature saturation.

**Figure 1:**
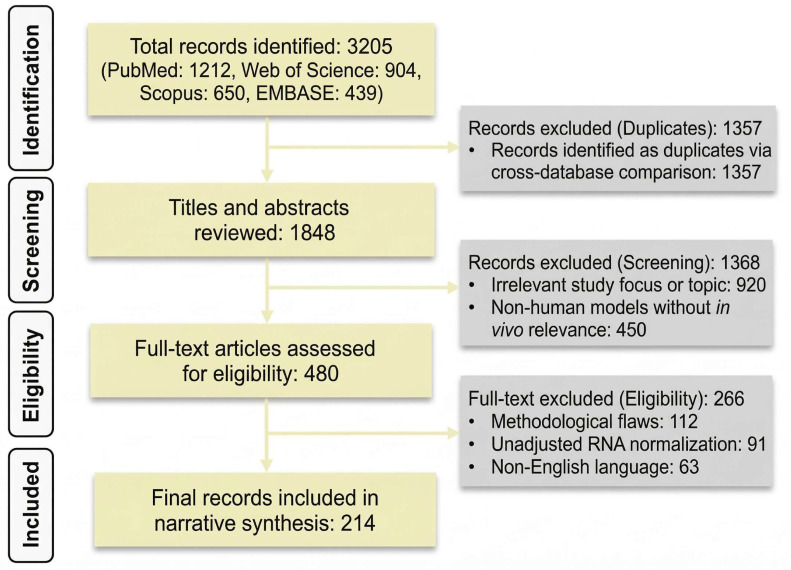
Literature selection flow diagram.

The quantitative flow of the literature search is summarised in Figure 1. Initial database queries returned approximately 4217 records (PubMed/MEDLINE ≈ 2108; Web of Science ≈ 1394; Scopus ≈ 504; Embase ≈ 211). After deduplication (≈1096 records removed), 3121 records were screened by title and abstract; 2584 were excluded as off-topic, leaving 537 records for full-text evaluation. Of these, 218 met the inclusion criteria for primary citation; 12 additional records were retrieved through forward and backward citation tracking, yielding a final corpus of 230 cited sources. As Figure 1 makes apparent, the literature is heavily biased toward acute mTORC1 signalling: of the 537 full-text-evaluated records, only ≈14% reported direct human measurements of ribosomal-end variables (total RNA, 45S pre-rRNA, mature 18S/28S, or RNA:DNA ratio). This evidentiary asymmetry constitutes one of the principal research gaps motivating the present synthesis.

Three closely related but conceptually distinct terms recur throughout the manuscript and warrant explicit definition. Ribosomal pool size refers to the standing stock of mature 80S ribosomes in the muscle fibre and is quantified empirically by total RNA content per unit of tissue or, more precisely, by mature 18S/28S rRNA abundance per unit of cellular reference (e.g., DNA). Ribosome biogenesis refers to the dynamic process by which this pool is renewed and expanded, encompassing rDNA transcription by RNA Polymerase I, generation and processing of the 45S pre-rRNA precursor, ribosomal-protein synthesis, and nucleolar assembly of the mature subunits. Translational capacity is the functional outcome of pool size: the maximal aggregate rate at which a fibre can synthesise protein given saturating mechanical and nutritional input. In this hierarchy, biogenesis is the kinetic input, ribosomal pool size is the structural state variable, and translational capacity is the functional consequence.

The inclusion criteria were strictly defined to prioritize translational and physiological relevance. Eligible publications encompassed peer-reviewed original research, clinical trials, and foundational preclinical models that directly quantified markers of ribosome biogenesis (e.g., total RNA accumulation, mature 18S/28S rRNA fractions, precursor 45S transcripts, or nucleolar initiation factors, such as c-Myc, UBF, and TIF-1A) in response to loading or unloading stimuli. Human in vivo studies—particularly longitudinal resistance training interventions, detraining models, and aging cohorts—were heavily prioritized to establish the concept of translational capacity as a definitive phenotypic bottleneck. Animal and sophisticated in vitro models (e.g., synergistic ablation, single-fiber mechanobiology) were selectively included only when they provided indispensable mechanistic insights into nuclear mechanotransduction or ribosomal turnover kinetics that are currently impossible to resolve in living humans.

Rigorous exclusion criteria were applied to maintain strict conceptual boundaries. Studies focusing exclusively on congenital ribosomopathies, cancer-derived cell lines without direct relevance to skeletal muscle plasticity, or pharmacological interventions isolated from exercise or targeted nutritional physiology were categorically discarded. Furthermore, non-peer-reviewed materials, conference abstracts, and editorials lacking robust empirical synthesis were excluded. We also critically evaluated the internal validity of the included molecular studies; while articles utilizing highly confounded RNA normalization strategies (such as unadjusted wet-tissue weight) were not entirely excluded, their methodological limitations are explicitly addressed and contextualized within the review.

The final body of literature was screened iteratively, first by title and abstract, followed by a rigorous full-text methodological evaluation to ensure alignment with the review’s primary objectives. The complete literature search, screening phases, and selection process, detailing the progression from initial record identification to final rigorous inclusion, are visually summarized in the flow diagram in Figure 1.

## 3. Cellular and Molecular Foundations of Ribosome Biogenesis

Understanding ribosome biogenesis in skeletal muscle requires moving beyond isolated molecular events and considering how structural, signaling, and energetic processes interact to regulate translational capacity. Although acute signaling pathways can transiently influence protein synthesis, sustained hypertrophy depends on coordinated changes in ribosomal content, nucleolar activity, and gene expression. The following sections examine these processes in a structured manner, from key conceptual distinctions to the regulation of nucleolar function and the constraints that ultimately shape translational capacity.

### 3.1. Translational Efficiency vs. Translational Capacity

To establish translational capacity as the primary bottleneck for skeletal muscle hypertrophy, it is essential to distinguish between translational efficiency and translational capacity, two concepts that are often conflated. Translational efficiency refers to the rate at which existing ribosomes translate mRNA into polypeptides, a process largely governed by transient changes in mTORC1 signaling [53]. In contrast, translational capacity reflects the total number of functional ribosomes within the sarcoplasm and therefore defines the structural potential for sustained protein synthesis over time [20].

**Figure 2:**
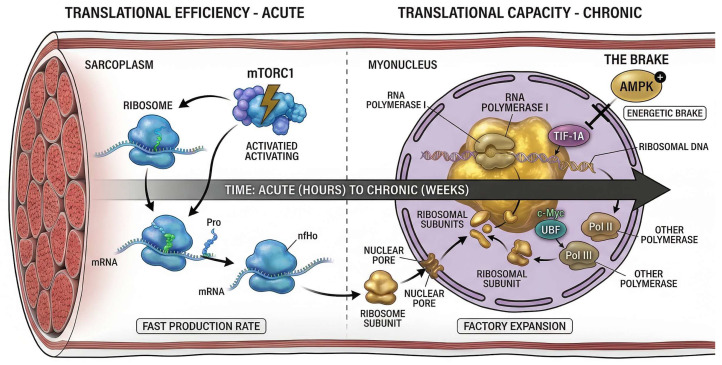
Conceptual schematic differentiating translational efficiency from capacity. Mechanical loading stimulates mTORC1 to rapidly increase the translation rate at existing ribosomes (Efficiency, acute phase). Simultaneously, parallel signaling cascades involving c-Myc, UBF, and TIF-1A activate RNA Polymerases I, II, and III in the nucleus and nucleolus to synthesize new ribosomal subunits (Capacity, chronic phase). AMPK acts as an energetic brake, inhibiting TIF-1A to suppress ribosome biogenesis during metabolic stress. Importantly, both stretch loading (e.g., eccentric training) and pharmacological anabolic stimuli (e.g., anabolic–androgenic steroids) act on both branches of this diagram: in addition to amplifying mTORC1-driven translational efficiency, they have been shown to increase ribosomal biogenesis through Pol I activation and c-Myc/UBF/TIF-1A signalling, expanding translational capacity over time.

Recognizing ribosome biogenesis as the key mechanism underlying increases in translational capacity shifts the focus away from short-lived signaling responses and toward longer-term adaptations in cellular infrastructure (Figure 2). This paradigm shift suggests that while acute spikes in protein synthesis are necessary for immediate repair, they are ultimately constrained by the fixed “ceiling” of the existing ribosomal pool. To achieve significant phenotypic growth, the muscle fiber must prioritize the expansion of its protein-synthetic machinery over the mere acceleration of its current activity.

Furthermore, this distinction carries profound implications for understanding the diminishing returns observed in elite trainees. In the early stages of hypertrophy, improvements in translational efficiency may suffice to drive adaptation; however, as the muscle approaches its physiological limit, the relative importance of translational capacity becomes paramount. Without a concomitant increase in the number of ribosomes, even the most potent anabolic stimuli will fail to translate into further gains, as the system becomes physically saturated at the translational level.

### 3.2. Nucleolar Structure and Ribosome Biogenesis in Skeletal Muscle

The nucleolus in skeletal muscle is a highly organized nuclear structure rather than a diffuse accumulation of ribonucleoproteins. Its tripartite organization reflects the sequential stages of ribosome production and is sensitive to both mechanical and metabolic signals [54]. Ribosomal DNA (rDNA) loci, arranged in tandem repeats on acrocentric chromosomes, are transcribed within this compartment. The initial processing of the 45S pre-rRNA transcript takes place in the dense fibrillar component (DFC), while later maturation steps and assembly with ribosomal proteins (RPs) and 5S rRNA occur in the granular component (GC), prior to export to the cytoplasm [5,30].

The synthesis of a functional 80S ribosome is one of the most energy-demanding processes in the muscle fiber and requires the coordinated activity of all three nuclear RNA polymerases [5,23]. RNA polymerase I drives transcription of the 47S/45S precursor, whereas RNA polymerase II produces ribosomal proteins and RNA polymerase III generates 5S rRNA and transfer RNAs. This coordination is essential: activation of mTORC1 alone is not sufficient if the outputs of the three polymerases are not synchronized, reinforcing the idea that ribosome biogenesis is a tightly integrated process [23].

Beyond its role as a factory, the nucleolus functions as a sophisticated molecular sensor that integrates mechanical tension with nutrient availability. The expansion of nucleolar volume and the reorganization of its internal architecture serve as early morphological hallmarks of a fiber transitioning into a pro-anabolic state. This structural plasticity allows the myofiber to rapidly ramp up its biosynthetic potential in response to localized stress, ensuring that the supply of new ribosomes meets the increased demand for contractile protein synthesis.

### 3.3. Molecular Regulation of Ribosomal Assembly

The maturation of ribosomal subunits from the 45S precursor involves a complex processing cascade mediated by small nucleolar RNAs (snoRNAs) [55]. These molecules guide site-specific modifications, including 2′-O-methylation and pseudouridylation, which are necessary for correct ribosome structure and function [32,55]. This process is not static; evidence indicates that snoRNA expression increases in response to resistance exercise, suggesting that processing capacity must adapt alongside transcriptional output [55].

At the same time, ribosomal proteins synthesized in the cytoplasm are transported into the nucleus and incorporated into assembling ribosomal subunits within the nucleolus [56]. The coordination between ribosomal protein availability and rRNA processing is critical to ensure efficient assembly and to prevent degradation of unprotected RNA intermediates [5,56]. Only fully assembled pre-40S and pre-60S subunits are exported to the cytoplasm, where they combine to form functional ribosomes [30,56].

This intricate assembly line is subject to rigorous quality control mechanisms that monitor the integrity of the ribosomal subunits before they reach the sarcoplasm. Any imbalance between the supply of ribosomal proteins and the transcription of rRNA can lead to nucleolar stress, potentially triggering signaling pathways that halt the biogenic process. Consequently, the efficiency of ribosomal assembly serves as a secondary bottleneck, ensuring that the fiber only invests energy in the production of high-fidelity translation machinery capable of sustained hypermetabolic activity.

### 3.4. mTORC1 and the Initiation of Ribosome Biogenesis

Ribosome biogenesis is closely linked to nutrient and growth factor signaling, with mTORC1 acting as a central regulator [15]. In addition to its role in translational efficiency, mTORC1 influences nucleolar activity by promoting transcription of ribosomal RNA. One key mechanism involves phosphorylation of the transcription factor TIF-1A, which facilitates its interaction with RNA polymerase I and enhances transcription of the 45S precursor [28,57].

**Figure 3:**
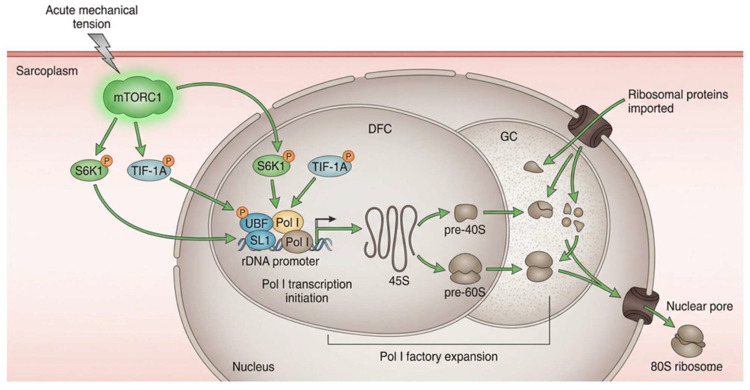
Detailed molecular mechanisms linking mTORC1 activation to nucleolar ribosome biogenesis. This figure integrates mechanical tension from the sarcoplasm to Pol I initiation within the granular component (GC). Acute mechanical tension (encompassing concentric, isometric, and stretch/eccentric loading; *lightning bolt*) activates mTORC1, which phosphorylates S6K1 and TIF-1A (*illustrated by orange dots*). The phosphorylated effectors enter the dense fibrillar component (DFC) to phosphorylate and activate the transcription factors UBF and SL1, recruiting RNA Pol I to the rDNA promoter (curled DNA structure). This drives the detailed, step-by-step processing from the massive 45S pre-rRNA transcript to mature subunits. As pre-40S and pre-60S subunits mature in the DFC, they move to the granular component (GC) to assemble with ribosomal proteins imported from the sarcoplasm. Only fully formed mature 80S ribosomes are exported into the cytoplasm through nuclear pores (dark brown structures) to join the expanded translating pool. The entire factory is labeled ‘Pol I factory expansion’ to represent translational capacity expansion.

mTORC1 also activates p70S6K1, which in turn phosphorylates upstream binding factor (UBF), a protein required for recruitment of RNA polymerase I to rDNA promoters [24,57]. These events increase accessibility of rDNA regions by modifying chromatin structure [29]. Together, these mechanisms illustrate how acute mechanical signals are translated into sustained transcriptional responses that support ribosomal expansion (Figure 3).

### 3.5. c-Myc and the Transmission of the Mechanical Signal

Mechanical loading is also conveyed to the nucleolus through the transcription factor c-Myc [32,58]. Resistance exercise induces a rapid increase in c-Myc expression in skeletal muscle, typically within a few hours after the stimulus [32,59]. c-Myc acts broadly by enhancing transcriptional activity across RNA polymerases I, II, and III, thereby coordinating multiple steps of ribosome biogenesis [32]. Stretch overload, including the eccentric component of resistance training and synergist-ablation models in preclinical work, has also been reported to induce c-Myc expression, indicating that the c-Myc/Pol I axis is responsive to both shortening and lengthening contractile actions.

Importantly, c-Myc activation is not entirely dependent on mTORC1. Mechanical signals can be transmitted directly to the nucleus through the LINC complex, facilitating c-Myc translocation independently of cytoplasmic signaling pathways [60]. This may explain why short-term increases in protein synthesis do not always predict long-term hypertrophy: without sufficient induction of c-Myc, the expansion of translational capacity may be limited despite favorable metabolic conditions [58,60].

### 3.6. AMPK and the Energetic Regulation of Ribosome Biogenesis

Ribosome biogenesis is also constrained by cellular energy availability, primarily through the activity of AMPK [23]. While AMPK is known to inhibit mTORC1 and reduce translational efficiency, it also directly suppresses ribosomal RNA synthesis [30,61]. This occurs under conditions of high metabolic stress, such as intense or prolonged resistance exercise.

AMPK can inhibit RNA polymerase I activity through multiple mechanisms, including suppression of TIF-1A function and modulation of chromatin accessibility at rDNA loci [61,62,63]. As a result, there is a balance between mechanical signals that promote ribosome production and energetic signals that limit it. This “metabolic brake” serves as a protective mechanism to prevent the myofiber from entering an energetic crisis during periods of extreme demand. If the metabolic cost of building new ribosomes exceeds the available ATP supply, AMPK-mediated repression takes precedence, effectively halting the expansion of translational capacity; under such conditions, mitochondrial ATP availability functionally becomes a co-limiting step for translational-capacity expansion, even when mechanical input and amino-acid supply are otherwise adequate.

Understanding this antagonism is crucial for optimizing training programs, as it defines the upper limit of volume beyond which the stimulus for ribosome biogenesis is neutralized by the stress of the exercise itself.

### 3.7. Ribosome Turnover and the Limits of Translational Capacity

Translational capacity is determined not only by the rate of ribosome synthesis but also by the rate of ribosome degradation [34]. Ribosomes are relatively stable structures, with half-lives that can extend over several days in skeletal muscle [5,25]. This stability means that increases in ribosomal content persist beyond the initial stimulus but also require ongoing maintenance.

Ribosome degradation is mediated in part by ribophagy, a selective form of autophagy [64]. Under conditions such as disuse or energy restriction, ribophagy can reduce ribosomal content and limit translational capacity. In trained individuals, repeated exposure to similar stimuli may attenuate the transcriptional response associated with ribosome biogenesis, making further increases in capacity more difficult to achieve [65].

### 3.8. Satellite Cells and Myonuclear Addition

The long-term expansion of translational capacity may ultimately be constrained by the myonuclear domain (MND), which defines the cytoplasmic territory supported by each myonucleus [22]. In early or moderate phases of hypertrophy, some degree of growth may occur without immediate satellite cell fusion, provided that existing myonuclei retain sufficient transcriptional reserve [22]. However, as muscle fibers continue to adapt to repeated mechanical loading, the capacity of the pre-existing nuclear population may become increasingly limiting. Under these conditions, further expansion of ribosome biogenesis and protein synthetic infrastructure may depend, at least in part, on the incorporation of additional myonuclei [66].

This possibility places satellite cells at the center of long-term architectural remodeling. Following activation, proliferation, and fusion, Pax7+ satellite cells donate new myonuclei to the syncytium, thereby expanding the transcriptional and nucleolar infrastructure available to support continued adaptation [25,66,67]. Rather than acting as an obligatory requirement at all stages of hypertrophy, satellite cell fusion is better interpreted as a mechanism that becomes increasingly relevant when sustained loading pushes existing myonuclei toward their functional limits [22,68]. In this framework, myonuclear addition may help preserve the capacity for continued ribosomal accretion during prolonged training periods.

**Figure 4:**
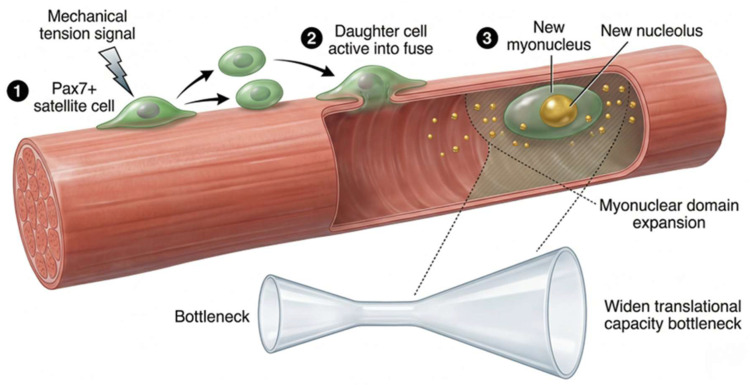
Satellite cell fusion and myonuclear domain expansion to permanently widen the translational capacity bottleneck. The conceptual diagram illustrates how the muscle fiber overcomes the adaptive ceiling experienced by advanced athletes. Mechanical tension (*lightning bolt*) activates Pax7+ satellite cells (*green*), which proliferate and fuse with the existing syncytium. This fusion process donates a new myonucleus and, critically, a new nucleolar factory template to the syncytium. By increasing total nucleolar mass, the fiber expands its individual myonuclear domain (MND) and permanently widens the architectural reserve required to widen translational capacity (visualized as a widening funnel). This integration of new machinery breaks the bottleneck saturation, permitting a new phase of accelerated phenotypic hypertrophy.

This perspective may also help explain why hypertrophic progress often slows in highly trained individuals. Once the initial gains supported by existing myonuclei have been largely realized, further adaptation may require a slower phase of structural remodeling involving satellite cell participation and myonuclear expansion [66,68]. Importantly, nuclei acquired during training may persist during subsequent unloading, contributing to the cellular basis of muscle memory and facilitating a more rapid re-expansion of translational capacity during retraining [67,69] (Figure 4). Thus, satellite cells should be viewed not as a constant driver of all hypertrophy, but as a potentially important contributor to the long-term maintenance and expansion of the translational machinery.

## 4. Common Methodological Issues in Assessment of Ribosomal Biogenesis

Assessing ribosome biogenesis in living human skeletal muscle presents profound methodological hurdles that frequently obscure the entire mechanistic continuum from mechanotransduction to phenotypic adaptation [20,23,30]. Unlike transgenic animal models, where absolute ribosomal synthesis rates can be directly resolved using dynamic metabolic labeling techniques (e.g., stable isotope tracing or radioactive precursors), human trials are largely constrained to static snapshots derived from invasive percutaneous needle biopsies [34,70]. This fundamental spatial and temporal bottleneck necessitates a critical and rigorous evaluation of contemporary methodologies, as reliance on reductionist bulk tissue assays and flawed mathematical interpretations has systematically confounded the applied hypertrophy literature for decades [33,37]. To definitively widen the translational bottleneck, research must transition toward spatially precise, metabolically traceable methodologies that resolve absolute infrastructural expansion [49,71].

### 4.1. Bulk RNA Quantification: Cellular Infiltration and Technical Limitations

In human clinical and sports science trials, the quantification of total bulk RNA extracted from muscle homogenates serves as the ubiquitous primary proxy for cellular ribosomal density [36,49]. The biochemical justification is robust: stable ribosomal RNA (rRNA) species (specifically, the massive 28S and 18S subunits) constitute approximately 80% to 85% of the entire intracellular RNA pool [5,23]. Consequently, a statistically significant accumulation of bulk total RNA following a standardized mechanical stimulus, such as exhaustive resistance exercise, is robustly interpreted as an expansion of the mature rRNA pool and, thus, a definitive widening of the translational capacity bottleneck [33,35]. This foundational correlation remains statistically strong [15,17] and has been corroborated by seminal work validating RNA accumulation alongside MPS kinetics [16,70].

However, this ubiquitous bulk assay masks critical biological and spatial bottlenecks. First, bulk tissue homogenization inherently blends the transcriptomic profiles of multinucleated myofibers with the RNA derived from resident non-muscle cells, including fibroblasts, endothelial cells, infiltrating immune cells (macrophages and neutrophils), and uncommitted satellite cells [48,72]. If a high-volume mechanical stimulus induces severe microtrauma, the subsequent inflammatory response can drastically increase the non-muscle cell population within the biopsy sample, falsely elevating the bulk total RNA concentration [48,73]. This cellular infiltration confounder can lead to systemic false-positive interpretations of muscle-specific translational capacity expansion, effectively obscuring the true magnitude of the nucleolar output driving myofibrillar hypertrophy [72,73].

Furthermore, bulk total RNA quantification offers only a static, time-averaged assessment of cellular infrastructure and cannot differentiate between a rapid, transient spike in transcriptional initiation and the actual, slow accumulation of mature ribosomal subunits [5,34]. As mature mammalian ribosomes are exceptionally stable, with estimated in vivo half-lives spanning several days to over a week [25,64], total RNA accumulation lags significantly behind the immediate activation of RNA polymerase I [34]. Standardized biopsy timing is mandatory for practitioners attempting to capture the initial widening of the structural bottleneck. A biopsy taken too early or too late may completely miss the critical 45S pre-rRNA transcript peak or the subsequent mature rRNA accumulation. This methodological error leads to the erroneous conclusion that nucleolar initiation did not occur, a temporal pitfall that is particularly evident when studying age-related anabolic resistance and cumulative ribosomal deficits [14,55].

### 4.2. qPCR and Specific Transcripts in the Study of Ribosomal Biogenesis

To resolve the temporal limitations of total RNA, researchers frequently utilize quantitative PCR (qPCR) to target specific transcriptional intermediates within the nucleolar assembly line, particularly the massive 45S pre-rRNA precursor [55]. Because this 45S transcript is rapidly processed and possesses a half-life measured in minutes, its abundance serves as an ultrasensitive, real-time indicator of active Pol I initiation and nucleolar stress [30,58]. Acute human resistance exercise potently induces rapid spikes in 45S pre-rRNA expression, peaking typically between 2 and 4 h post-stimulus, often returning to basal levels within 24 to 48 h, providing conclusive evidence that mechanical loading effectively initiates the widening of the structural bottleneck [14,55]. Importantly, this transcriptional spark is not, by itself, sufficient: under chronic overtraining conditions, repeated Pol I activation can fail to translate into net translational-capacity expansion because persistent AMPK activation, nucleolar stress, and accelerated ribophagy shift the balance toward net rRNA loss; in this scenario, MPS rates can plateau or even decline despite preserved or augmented acute 45S pre-rRNA responses, suggesting that overstimulation can functionally reverse the long-term anabolic yield of repeated Pol I spikes.

However, this transcript-specific approach is fundamentally reductionist and biologically vulnerable to unmeasured bottlenecks [57]. An elevated abundance of the 45S pre-rRNA intermediate does not obligatorily result in the assembly of mature, functional ribosomal subunits; Pol I initiation does not guarantee complete maturation [32,33]. qPCR only measures transcript abundance; it cannot assess the dynamic synchronization of all three RNA polymerases (Pol I, II, and III) required for stoichiometric assembly, nor can it capture the architectural complexity of nucleolar processing [23,57]. A novel biomechanical stimulus might successfully initiate Pol I, but concurrent metabolic stress might abort snoRNA-guided processing downstream, leading to aborted assembly despite massive precursor transcripts [60,62].

A critical limitation of qPCR in this context is the discordant regulation of ribosomal protein (RP) mRNAs [74]. The mRNAs encoding the ~80 ribosomal proteins possess a unique 5′ terminal oligopyrimidine (5′ TOP) tract, which strictly regulates their translation via the mTORC1/eIF4E signaling axis [74,75]. qPCR can quantify the abundance of these RP mRNAs transcribed by Pol II, but it cannot confirm whether these transcripts are actively being translated by existing ribosomes in the sarcoplasm [75]. If mTORC1 is inhibited by energetic stress, RP mRNAs may accumulate in the cytoplasm without being translated, creating a stoichiometric deficit that halts the entire assembly of the 80S ribosome, a failure that is invisible to standard qPCR methodologies [74].

Similarly, quantifying small nucleolar RNAs (snoRNAs) responsible for guiding the modification of the 45S precursor presents severe technical artifacts [55]. Many snoRNAs are deeply nested within the introns of host genes (often RP genes) and are excised during splicing [76]. Extracting and quantifying these small, highly structured RNAs from bulk muscle biopsies requires specialized small-RNA-specific extraction columns and modified reverse-transcription protocols [76]. Standard total RNA extraction kits frequently lose these low-molecular-weight species, leading to severe underestimations of processing capacity [55,76]. Consequently, relying solely on standard qPCR to assess the complete biogenesis pipeline is fraught with technical and biological pitfalls.

### 4.3. Challenges of RNA Normalization in Ribosomal Biogenesis Research

It is important not to overstate the methodological case in either direction. The RNA:DNA ratio is biologically attractive because it controls for cellularity, but it is itself ambiguous in two specific scenarios that are highly relevant to skeletal muscle research. First, a rising RNA:DNA ratio cannot a priori distinguish a genuine expansion of the ribosomal pool from a reduction in myonuclear or stromal DNA. Second, in longitudinal resistance-training studies the very satellite-cell fusion that we have argued is functionally important for late-phase hypertrophy alters the denominator by adding new myonuclei, which can mask further pool expansion. RNA:DNA is therefore not a universally superior method but a useful adjunct whose interpretation depends on the experimental question and timeframe [37,68]. Wet-weight normalisation is biologically blunt—particularly when oedema and intracellular fluid shifts are present after intense training [50]—but is not without value in stable resting tissue. The most defensible approach is methodological pluralism: report total RNA per unit wet weight, total RNA per unit DNA, and absolute 45S pre-rRNA and mature rRNA species in parallel, and interpret each against the specific question being asked. Much of the apparent inconsistency in the prior literature can be traced not to one method being wrong but to authors selecting a single denominator without acknowledging the assumptions it implies [37,77].

Perhaps the most pervasive and consequential methodological flaw plaguing the contemporary human hypertrophy literature is the mathematical normalization of bulk RNA data [37]. Traditionally, total RNA or specific transcripts have been reported relative to the initial wet weight of the muscle biopsy tissue sample (e.g., nanograms of RNA per milligram of wet tissue). This standard laboratory practice makes a profound and flawed biological assumption: that the denominator (wet tissue mass) remains dynamically stable during actively growing tissue [37,50]. Resistance exercise induces sarcoplasmic hypertrophy, characterized by a disproportionate expansion of intracellular fluid, glycogen, and soluble proteins occurring at a much faster rate than dense myofibrillar accretion [37,50]. This rapid volumetric expansion dilutes the apparent concentration of RNA within the sarcoplasm, creating the pervasive moving denominator problem.

**Figure 5:**
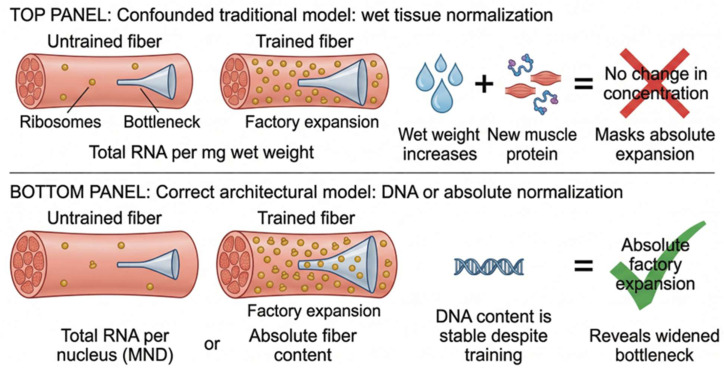
The moving denominator paradox in human bulk muscle RNA normalization. The diagram contrasts how flawed mathematical interpretation of bulk tissue data can mask true architectural expansion. Top panel illustrates an untrained fiber (low ribosome density, narrow bottleneck funnel) versus a trained fiber (expanded factory, golden ribosomes added, wider bottleneck). When bulk homogenate RNA is mathematically normalized to total wet tissue weight (which increases via growth, edema, and protein), the result appears as ‘no change in concentration’, masking the actual absolute expansion. Bottom panel illustrates the same expansion: when bulk RNA is normalized to stable DNA content (nuclei) or measured as absolute fiber content, the true widening of the bottleneck funnel and factory expansion are revealed.

The moving denominator paradox perfectly elucidates why numerous longitudinal human trials have historically concluded that ribosome biogenesis is “not required” for phenotypic hypertrophy [37]. If a muscle fiber successfully adapts to mechanical loading by synthesizing new contractile proteins and new ribosomes at the same volumetric rate (maintaining constant physical density), the mathematical ratio of RNA per milligram of tissue will remain perfectly static. Relying solely on wet-tissue normalization would erroneously conclude that translational capacity did not expand, when the absolute physical number of ribosomes per fiber has dramatically increased [37,50]. This methodological blind spot systematically underestimates the necessity of ribogenesis, effectively closing the molecular bottleneck in the literature through flawed mathematical interpretation and reductionist normalization strategies (Figure 5).

To definitively resolve this moving denominator bottleneck, modern Q1 methodology demands alternative, mathematically stable normalization strategies [37]. Normalizing total RNA abundance to stable DNA content (generating an RNA:DNA ratio) provides a substantially more accurate reflection of translational capacity expansion [77]. Because the DNA content represents the total number of myonuclei—assuming satellite cell fusion is accounted for—an increasing RNA:DNA ratio explicitly demonstrates that existing individual nucleoli are successfully upregulating their transcription and expanding their individual factory output [77]. Alternatively, emerging isolated single-fiber transcriptomics, which report absolute RNA content per isolated myofiber segment, entirely bypasses the tissue-denominator problem [68]. By implementing these rigorous normalization techniques, researchers can definitively reveal the true architectural expansion driving human muscle hypertrophy.

### 4.4. Spatial and Volumetric Constraints in Nucleolar Imaging

Resolving the architectural expansion required to widen the translational capacity bottleneck requires direct visualization of nucleolar dynamics; however, human muscle imaging is currently limited by severe spatial and volumetric constraints [23,78]. Traditional imaging approaches in muscle biology have historically relied on 2D percutaneous needle biopsies analyzed via transmission electron microscopy (TEM) to resolve nucleolar density and area [30]. Although TEM provides unmatched ultrastructural resolution, it offers only an ephemeral snapshot of localized, non-homogeneous nucleolar structures that may not reflect the entirety of a multinucleated syncytium [30,78]. For advanced Q1 research, 2D surface area measurements must transition toward volumetric 3D analysis using confocal or super-resolution microscopy (e.g., STED or STORM) to accurately quantify the expansion of the nucleolar volume [54].

To move beyond mere structural volume, Q1 methodology is increasingly deploying multiplexed immunofluorescence and fluorescence in situ hybridization (FISH) to map the functional flow of biogenesis [79]. By simultaneously tagging Pol I (initiation), fibrillarin (processing in the DFC), and nucleophosmin (assembly in the GC), researchers can visualize whether the entire nucleolar assembly line is expanding or if a specific sub-compartment is bottlenecked [79,80]. However, the rigorous optimization required for human muscle tissue autofluorescence mitigation, combined with the high technological barrier of super-resolution platforms, creates significant infrastructural limitations, leaving the literature fragmented [78,79].

Table 1 summarizes the contemporary imaging and spatial techniques utilized to resolve nucleolar dynamics, highlighting the critical trade-offs between resolution, spatial context, and molecular specificity. Understanding these modalities is essential for interpreting the varying degrees of structural evidence presented in the hypertrophy literature [78,79,80].

### 4.5. Dynamic Omics Models of Ribosomal Biogenesis

To permanently alleviate the methodological bottleneck, the assessment of ribosome biogenesis must transcend reductionist bulk assays and static transcriptomic snapshots, evolving toward metabolically traceable and globally synchronous “omics” architectures [23,49,71]. While the integration of whole-muscle RNA sequencing (RNA-seq) has successfully mapped the global transcriptomic landscape of exercise adaptation—revealing that snoRNAs and Pol II-derived ribosomal proteins are dynamically synchronized with Pol I activity—this approach remains hindered by profound cellular infiltration confounders [33,48,55]. The definitive frontier lies in single-nucleus RNA sequencing (snRNA-seq), which permits the high-resolution isolation of nucleolar output specifically from myofiber-derived myonuclei, effectively decoupling the analysis from non-muscle cellular noise, such as infiltrating immune cells and fibroblasts [49].

Despite its cellular precision, snRNA-seq necessitates the destruction of the fiber’s spatial architecture. To resolve the functional “geography” of ribosome biogenesis, advanced Q1 methodology is increasingly deploying spatial transcriptomics (e.g., visium or slide-seq) to map mRNA and pre-rRNA transcripts directly onto intact histological sections [81,82]. This spatial resolution is critical to determine whether subsarcolemmal nuclei upregulate biogenesis with greater kinetic urgency than intermyofibrillar nuclei, or how distinct functional clusters (such as specific innervation-responsive zones) locally upregulate their ribogenic response, thereby adding a crucial topological dimension to the bottleneck theory [81,82].

Finally, the inherent limitations of the static snapshot must be overcome through the adoption of metabolically traceable human models. The integration of dynamic metabolic labeling using deuterium oxide (D_2_O) allows researchers to quantify the fractional synthetic rate (FSR) and decay kinetics of ribosomes in human muscle biopsies over longitudinal training periods [70,71]. When coupled with high-resolution mass spectrometry to track ribosomal protein turnover, D_2_O labeling directly resolves the absolute infrastructural expansion of the ribosomal pool with temporal precision [70,71]. By synergizing spatially resolved (spatial transcriptomics) and metabolically dynamic (D_2_O FSR) frameworks, future Q1 research can definitively circumvent current normalization bottlenecks and elucidate the true kinetic drivers of human muscle hypertrophy.

## 5. In Vivo Human Evidence: Ribosome Biogenesis in Phenotypic Adaptation

### 5.1. Early Transcriptomic Responses During Acute Exercise Exposure

Translating the elegant molecular orchestration of the nucleolus into applied sports science requires rigorous examination of in vivo human trials. While murine models utilizing synergistic ablation offer profound mechanistic insights, the extreme, non-physiological nature of such surgical overload cannot faithfully replicate the transcriptomic kinetics induced by voluntary resistance exercise in humans [83,84]. Consequently, contemporary human evidence relies on repeated percutaneous muscle biopsies to track the temporal dynamics of the nucleolar response. The foundational premise of the bottleneck theory is that mechanical tension must be converted into a rapid transcriptomic “spark” before any physical expansion of the ribosomal pool can occur, a process entirely dependent on the immediate activation of RNA Polymerase I (Pol I) [32].

In humans, this acute response is predominantly captured by quantifying the expression of the 45S pre-rRNA transcript and the global amplifier c-Myc. In a seminal human trial, Figueiredo et al. mapped this acute time course, demonstrating that following a single bout of heavy resistance exercise, c-Myc mRNA expression surges by over 10-fold within the first 2–4 h [85]. This massive c-Myc induction precedes and obligatorily drives a subsequent, highly significant elevation in 45S pre-rRNA, which typically peaks between 4 and 12 h post-exercise before gradually returning to baseline [85,86]. Crucially, contemporary molecular models demonstrate that this c-Myc-driven nucleolar expansion and subsequent protein synthesis can operate entirely independently of mTORC1 activation [59].

The magnitude of acute Pol I initiation is also acutely sensitive to the mechanical dose, specifically the training volume. Hammarström et al. elegantly demonstrated this dose–response relationship in a within-subject human trial comparing low-volume (1 set) versus high-volume (3 sets) resistance exercise [35]. Their data revealed that while both protocols activated mTORC1 equivalently, only the high-volume protocol elicited a sustained, massive upregulation of total RNA and mature rRNA species over the ensuing weeks. This strongly indicates that the translational capacity bottleneck possesses a high activation threshold: a minimal mechanical volume is strictly required to sustain the Pol I transcription spark long enough to outpace the basal decay rate of existing ribosomes [35,87,88].

However, the human nucleolus exhibits profound transcriptomic refractoriness, commonly referred to as the “repeated bout effect” at the molecular level. Ogasawara et al. observed that the initial, massive spikes in ribosomal gene transcription seen in untrained individuals following a novel mechanical stimulus are severely blunted after several weeks of continuous training [65]. As the muscle fiber adapts and the initial structural bottleneck widens, an identical mechanical load (relative to the new 1RM) fails to elicit the same magnitude of 45S pre-rRNA expression [65,89]. This biological dampening forces advanced athletes to continuously manipulate external loading variables to overcome the newly expanded bottleneck and re-ignite the nucleolar spark [90].

Therefore, analyzing the acute transcriptomic spark reveals a fundamental physiological truth: the initial hours following resistance exercise are defined by preparation rather than synthesis. The transient spike in 45S pre-rRNA merely represents the *initiation* of new assembly lines, which alone is insufficient to drive macroscopic growth if the stimulus is not repeatedly applied [91]. The true biological bottleneck is not simply activating Pol I once, but sustaining this activation across multiple microcycles to permit the slow, steady accumulation of mature 80S ribosomes within the expanding sarcoplasm [91,92].

### 5.2. Long-Term Ribosomal Accretion and Variability in Adaptive Response

The transition from acute Pol I transcription to the chronic accumulation of mature ribosomes dictates the trajectory of macroscopic hypertrophy. In a landmark longitudinal study, Brook et al. utilized deuterium oxide (D_2_O) tracing and serial biopsies to map this transition over 6 weeks of human resistance training [36]. They discovered a profound temporal mismatch: the vast majority of muscle protein synthesis (MPS) and total RNA accumulation occurred within the first 3 weeks of training, whereas measurable increases in muscle cross-sectional area (CSA) primarily materialized in the final weeks. This is consistent with the hypothesis that widening the translational capacity bottleneck (ribosomal accumulation) is a critical antecedent event strongly associated with structural phenotypic hypertrophy [36,93,94].

The most compelling human evidence supporting this bottleneck theory emerges from studies on inter-individual variability conducted using cluster analysis. Human subjects universally exhibit vast heterogeneity in hypertrophic outcomes following standardized training protocols, which are broadly categorized as extreme (high), moderate, and non-responders [95]. Bamman’s laboratory was among the first to molecularly profile these distinct clusters, revealing that high responders are not characterized by unique circulating hormone profiles or superior dietary protein intakes, but rather by distinct transcriptomic and cellular architectural adaptations [95]. Current cluster studies are frequently limited by small sample sizes (often fewer than 30 participants per cluster), which restricts the robustness of definitive mechanistic conclusions. Rather than proving direct causation, these molecular profiles suggest that high responders may possess a highly permissive upstream cellular phenotype that simultaneously drives both rRNA transcription and macroscopic protein accretion.

Early investigations into these responder clusters, spearheaded by Petrella et al., demonstrated that extreme hypertrophic responders exhibited a high capability for satellite cell activation and myonuclear addition compared to non-responders [66]. Because new myonuclei donate new nucleoli to the syncytium, this finding aligns with the requirement to physically expand the architectural space available for ribosome biogenesis [66,96].

Subsequent molecular profiling by Mobley, Haun, and Roberts provided the definitive link to ribosome biogenesis [70,97]. By analyzing the vastus lateralis of individuals who underwent 12 weeks of heavy resistance training, they demonstrated that extreme responders exhibited massive increases in their RNA:DNA ratio (a marker of true ribosomal expansion per nucleus), alongside significant total RNA accumulation [70,97]. Conversely, extreme non-responders failed to significantly elevate total RNA or rRNA levels. The magnitude of hypertrophy was almost perfectly predicted by the fiber’s ability to successfully accumulate new ribosomal machinery, solidifying nucleolar expansion as the ultimate determinant of the high-responder phenotype [70,98].

This divergence becomes even more profound when examining the acute signaling cascades of these non-responders. Thalacker-Mercer et al. demonstrated that non-responders often exhibit robust, and sometimes even superior, acute mTORC1 and p70S6K phosphorylation following exercise compared to high responders [99]. This apparent paradox shatters the traditional “translational efficiency” model: despite sending a massive anabolic signal to existing ribosomes, non-responders completely fail to convert this signal into nucleolar expansion [99]. Their capacity bottleneck remains rigidly closed, rendering high-efficiency signaling biochemically useless for long-term accretion (Table 2).

The failure of the non-responder nucleolus underscores that the capacity bottleneck is highly individualized and genetically influenced. While environmental factors, such as total training volume, can be titrated to force adaptation, some individuals possess a rigid transcriptomic ceiling that resists mechanical deformation [100,101]. For practitioners, recognizing this limitation mandates a paradigm shift: when a client fails to grow, the solution is rarely adding more dietary protein to stimulate mTORC1, but rather manipulating the mechanical stimulus specifically to force the nucleolar bottleneck to open.

### 5.3. Age-Related Anabolic Resistance and Altered Nucleolar Regulation

Aging represents the most profound naturally occurring model of impaired translational capacity, clinically manifesting as sarcopenia and “anabolic resistance” [46]. While early studies in human cohorts attributed age-related muscle wasting to chronic systemic inflammation or insulin resistance, contemporary human biopsy data confirm that the primary defect resides deep within the structural architecture of the muscle fiber [102,103]. The senescent skeletal muscle nucleolus becomes severely desensitized to mechanical tension, creating an extraordinarily rigid bottleneck that resists widening, thereby accelerating the loss of myofibrillar mass [102].

The basal transcriptomic landscape of older adults provides the first evidence of this structural decay. Studies by Kirby et al. [38] and Chaillou & Montiel-Rojas [104] report that resting, untrained older adults exhibit significantly depleted levels of bulk total RNA and specifically mature 28S and 18S rRNA species compared to their young counterparts. This basal decrement indicates a chronic failure in the homeostatic maintenance of the ribosomal pool. Because the absolute factory size is significantly reduced, senescent muscle fibers are unable to mount a rapid, high-volume synthetic response when faced with metabolic stress, rendering the tissue highly vulnerable to catabolic crises (e.g., brief periods of bed rest) [104,105].

When older adults are subjected to heavy resistance training, nucleolar defects become acutely apparent. Stec, Mayhew, and Bamman elegantly demonstrated that following a standardized bout of resistance exercise, older adults fail to upregulate c-Myc and 45S pre-rRNA transcription [47]. While young participants exhibited the classic rapid nucleolar spark required to initiate biogenesis, the older cohort demonstrated a flat transcriptomic response. Crucially, Dickinson and Fry established that this failure occurs despite normal or hyperactive acute mTORC1 signaling in the elderly following exercise [19]. This proves that age-related anabolic resistance is not a signaling deficiency (efficiency) but a catastrophic failure in mechanotransduction and Pol I initiation (capacity) [47,106].

The precise mechanism underlying this senescent nucleolar rigidity is actively debated; however, emerging evidence strongly suggests alterations in the nuclear envelope and chronic nucleolar stress [41]. As established in Section 3, mechanotransduction requires a rigid LINC complex to physically deform the nucleus and recruit c-Myc. In aged muscle, cytoskeletal stiffness and nuclear lamina disorganization severely impair physical signal transmission [107,108]. Furthermore, chronic low-grade systemic inflammation, termed “inflammaging”, has been shown by Snijders et al. to directly induce nucleolar stress, prioritizing the synthesis of stress-response proteins over the energy-demanding transcription of massive 45S pre-rRNA precursors [73,109]. This pro-inflammatory and oxidative milieu may further compromise ribosomal homeostasis by reducing the post-transcriptional stability of rRNA species and RP mRNAs: chronic oxidative stress is known to accelerate transcript degradation and impair the activity of nucleolar processing machinery, so that the senescent muscle nucleolus likely faces a double constraint—diminished Pol I-driven synthesis on one side and accelerated RNA turnover on the other.

This severe constriction of the bottleneck has profound implications for clinical exercise prescription. Because the aged nucleolus is highly resistant to standard loading, Breen and Phillips have hypothesized that older adults require a significantly higher relative mechanical threshold to elicit the same molecular spark observed in the young [46,110]. However, this necessity clashes with the impaired recovery kinetics of aged tissue. Therefore, overcoming the sarcopenic bottleneck requires highly periodized interventions that provide massive, unaccustomed mechanical tension to forcefully widen the nucleolus [47,110].

Ultimately, the human aging model confirms the bidirectional power of bottleneck theory. The loss of translational capacity not only prevents hypertrophy but also actively dictates the trajectory of atrophy [38]. Until clinical therapies can successfully restore the mechanosensitivity of the senescent nucleolus and re-ignite Pol I transcription, treatments aimed solely at spiking mTORC1 efficiency will continue to fall short in reversing sarcopenia [111,112].

### 5.4. Detraining, Muscle Memory, and Retention of Ribosomal Adaptations

The final pillar of human evidence supporting the capacity bottleneck theory emerges from the kinetics of detraining and the phenomenon of “muscle memory.” When a highly trained individual abruptly ceases resistance exercise, macroscopic atrophy occurs rapidly within weeks, driven largely by the shedding of sarcoplasmic fluid and a rapid downregulation of translational efficiency [63,113]. However, the foundational architecture built during the training phase, specifically the expanded myonuclear domain and physical nucleolar templates, exhibits remarkable persistence [114]. Factory operations are temporarily halted; however, the concrete walls of the expanded factory remain largely intact.

Studies by Bruusgaard et al. [115] and Kadi et al. [116] utilizing advanced single-fiber techniques demonstrated that prolonged unloading leads to a slow decay in total RNA, whereas newly acquired myonuclei (donated via satellite cell fusion) are not eliminated via apoptosis. These nuclei represent permanent architectural expansion. Consequently, when an individual resumes training, the muscle fiber bypasses the initial, slow, and energetically demanding process of satellite cell proliferation [115]. The pre-existing, expanded nucleolar templates are immediately available to rapidly transcribe massive amounts of 45S pre-rRNA, explaining the accelerated rate of “re-growth” universally observed in previously trained humans [115,117].

The most compelling, albeit preliminary, molecular validation of this capacity for persistence has come from the field of epigenetics. Seaborne et al. mapped the DNA methylome of human skeletal muscle across a rigorous training, detraining, and retraining protocol [118], albeit with a small sample size that warrants replication in larger cohorts. They discovered an exploratory epigenetic memory in which genes that regulate tissue structure and ribosomal RNA promoters underwent significant hypomethylation (activation) during the initial training phase. Crucially, this hypomethylated signature was preserved even after weeks of total detraining and macroscopic atrophy [119,120]. However, because the sample size in these early studies was small and statistically underpowered for broad epigenome-wide association analyses, these specific methylation patterns must be considered hypothesis-generating rather than definitive. The rDNA promoters appeared to remain structurally “open” and biochemically primed for Pol I initiation [118,120].

**Figure 6:**
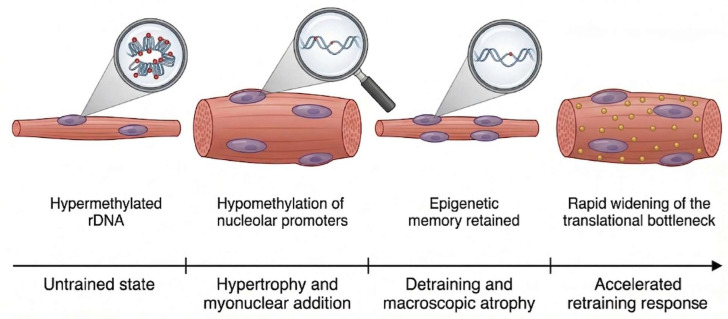
Cellular and epigenetic mechanisms underlying human muscle memory. The conceptual schematic illustrates the morphological and molecular progression of a muscle fiber through training, detraining, and retraining phases. During initial resistance training, satellite cell fusion donates new myonuclei, whereas rDNA promoters undergo significant hypomethylation (activation), permanently widening the nucleolar bottleneck. Following training cessation (detraining), the fiber experiences macroscopic atrophy due to a reduction in sarcoplasmic volume and ribosomal decay. However, the newly acquired myonuclei are strictly retained, and the rDNA promoters remain epigenetically primed (hypomethylated). Upon retraining, this persistent architectural and epigenetic memory completely bypasses the initial transcriptional bottleneck, allowing for an accelerated rate of ribosome biogenesis and rapid phenotypic regrowth.

This epigenetic uncoiling of the nucleolus provides definitive in vivo evidence for the bottleneck theory. The initial training phase opens the structural bottleneck, permanently altering the chromatin landscape of the rDNA loci [118,121]. Retraining does not require forcing a rigid bottleneck but rather sending the mechanical signal through a fully widened, epigenetically primed corridor. This facilitates an explosive re-accumulation of translational capacity, a process further optimized by early, fusion-independent communication from resident satellite cells that creates a highly permissive environment for long-term growth [122,123,124]. Thus, human muscle memory is fundamentally defined by the persistent epigenetic preservation of nucleolar expansion and primed MYC regulatory networks, ensuring faster adaptation upon subsequent loading [125,126,127] (Figure 6).

### 5.5. Concurrent Training, Interference, and Modulation of Ribosome Biogenesis

The integration of endurance and resistance training within the same periodized macrocycle, termed concurrent training, presents the ultimate in vivo human stress test for the translational capacity bottleneck [128]. Since Hickson’s foundational observations in 1980, sports scientists have recognized that high-volume endurance exercise blunts macroscopic strength and hypertrophy adaptations, a phenomenon known as the “interference effect” [128,129]. For decades, the molecular rationale for this interference was attributed exclusively to a conflict of translational efficiency: the energetic stress of endurance training activates AMP-activated protein kinase (AMPK), which subsequently phosphorylates TSC2 to directly inhibit mTORC1, thereby immediately halting acute muscle protein synthesis [129,130]. While this acute signaling conflict is biologically accurate, modern human evidence suggests that the true detriment of concurrent training lies not merely in transient signaling disruption, but in the sustained suppression of nucleolar expansion [131,132].

From a capacity bottleneck perspective, concurrent training imposes an important energetic limit on ribosome biogenesis [133]. The synthesis of a mature 80S ribosome requires the stoichiometric orchestration of three RNA polymerases and the transcription of thousands of massive pre-rRNA precursors, arguably making it the most ATP-demanding process within a muscle fiber [32,134]. It is important to acknowledge that AMPK is a highly pleiotropic kinase with essential, beneficial roles in skeletal muscle adaptation, including the promotion of mitochondrial biogenesis, glucose uptake, and cellular quality control. When exhaustive endurance exercise (such as high-intensity interval training or prolonged cycling) is performed in close temporal proximity to resistance training, the profound depletion of intramuscular glycogen and elevation of the AMP:ATP ratio robustly activate AMPK [134,135]. In this specific context of severe energy deficit, as established in foundational molecular models, AMPK acts as a severe nucleolar brake by suppressing the transcription initiation factor TIF-1A and actively restricting RNA polymerase I access to the rDNA promoter [131]. Therefore, the endurance stimulus substantially attenuates the transcriptomic spark required to widen the capacity bottleneck.

Human biopsy trials have elegantly captured this nucleolar sabotage. Fyfe, Hawley, and Stepto conducted rigorous in vivo investigations demonstrating that when human participants perform concurrent training, the acute post-exercise accumulation of specific ribosomal transcripts and nucleolar signaling cascades is significantly blunted compared to resistance training alone [129,136]. Similarly, meta-analytical data compiled by Wilson et al. confirm that the degree of macroscopic interference is highly dose-dependent, scaling primarily with the frequency and volume of the endurance modality [137]. If energetic stress is chronically high, the nucleolus remains perpetually constricted by AMPK, rendering the fiber entirely incapable of accumulating the total RNA necessary for long-term myofibrillar accretion, regardless of dietary protein ingestion [136,138]. The cell simply prioritizes mitochondrial survival over massive structural factory expansion.

However, human evidence reveals that the capacity bottleneck is highly sensitive to temporal recovery and modality selection. Seminal reviews by Baar and Murlasits have dictated that the molecular interference effect can be significantly mitigated if the energetic stress (AMPK activation) is spatially or temporally separated from the mechanical tension signal [138,139]. Human trials have shown that spacing endurance and resistance sessions by at least 6 to 24 h allows the acute nucleolar spark (c-Myc and 45S pre-rRNA) to initiate and stabilize before the catabolic, ATP-depleting endurance stimulus is introduced [138,139]. Thus, the concurrent training paradox ultimately proves that translational capacity is not merely a product of mechanical tension, but a highly sensitive energetic compromise. Overcoming the interference effect requires practitioners to precisely manage metabolic stress to ensure that the nucleolar bottleneck remains open long enough to successfully assemble the ribosomal machinery [133,139]. An anabolic-priority training sequence must be preserved.

From a practical standpoint, three operational rules emerge for athletes who must obtain the metabolic and cardiovascular benefits of endurance training without compromising ribosomal accretion. First, schedule endurance and hypertrophy-oriented resistance sessions on alternate days whenever programming permits, leaving the post-resistance anabolic window free of high-intensity endurance work for the ensuing 6–24 h. Second, when same-day training is unavoidable, perform the resistance bout first, recover with adequate protein and carbohydrate, and place the endurance session at the end of the day, ideally separated by ≥6 h. Third, prioritize lower-impact and lower-AMPK-activating endurance modalities (e.g., cycling, rowing, swimming) over prolonged running for athletes whose primary goal is hypertrophy, and bias endurance intensity toward moderate continuous work rather than exhaustive high-intensity intervals during dedicated hypertrophy blocks.

## 6. Practical Modulators of Ribosome Biogenesis

Ribosome biogenesis is one of the most plausible mechanisms linking repeated resistance-training exposure to sustained skeletal muscle hypertrophy. Its practical relevance depends not merely on whether a single session transiently alters molecular markers, but on whether the overall training, nutrition, and recovery environment repeatedly supports the transcription, processing, and accumulation of ribosomal material across a training block. From an applied perspective, the key issue is whether an intervention contributes meaningfully to the expansion or preservation of translational capacity over time, rather than simply elevating short-lived anabolic signaling after an isolated bout [29,140,141].

Accordingly, the most relevant practical modulators can be organized into three broad domains: training variables, nutritional variables, and a smaller set of supplemental or adjunct factors. In each case, the strength of inference should depend on the quality of the direct evidence available for ribosomal outcomes rather than on extrapolation from hypertrophy studies more generally. Where the literature only supports indirect conclusions, that uncertainty should be made explicit.

### 6.1. Training Variables

#### 6.1.1. Training Volume

Among training variables, weekly volume remains the factor with the clearest direct human support in relation to ribosome biogenesis. In the unilateral resistance-training study by Hammarström et al., participants who benefited most from the higher-volume condition were also those who showed the greatest early increases in total RNA, suggesting that ribosome accretion may help explain the superior adaptation sometimes seen with greater set volume [35]. This interpretation was reinforced by subsequent work showing that total RNA rises substantially during the early phase of a resistance-training block and declines with short-term training cessation, indicating that the ribosomal pool is highly plastic and closely linked to the continuity and magnitude of the loading stimulus [141].

Even so, volume should not be interpreted as a simple linear determinant of translational capacity. The broader hypertrophy literature demonstrates a dose–response relationship up to a certain threshold, beyond which adaptations plateau or regress due to overreaching [100,142]. The relevant issue is not only how many sets are performed, but whether that workload is recoverable. Too little volume may fail to create enough cumulative signaling to stimulate meaningful ribosome accumulation. Too much volume, in contrast, may increase local and systemic fatigue, reduce session quality, and compromise the energetic recovery conditions required to sustain ribosome biogenesis over time [142]. The applied implication is therefore not that more volume is always better, but that sufficient volume appears necessary, whereas excessive and poorly tolerated volume becomes self-limiting.

A further point is that the time course of ribosome biogenesis appears especially relevant during the early weeks of a training block. Hammarström et al. showed that total RNA accumulation was especially pronounced in the initial phase of training, suggesting that there may be a window in which skeletal muscle is particularly responsive in terms of ribosome accretion [141]. A moderate-to-high volume stimulus may be particularly productive when introduced during periods of renewed responsiveness, whereas the same absolute volume may have a smaller effect when imposed after prolonged training monotony. This reinforces the practical relevance of periodization even though the ribosomal literature is not yet mature enough to prescribe exact dose ranges with confidence.

#### 6.1.2. Load Intensity

Load intensity, understood as the relative percentage of one-repetition maximum (% 1RM), is another variable that deserves careful treatment. The broader hypertrophy literature consistently indicates that both low-load (e.g., 30% 1RM) and high-load (e.g., 80% 1RM) training can stimulate equivalent macroscopic muscle growth when sets are performed with sufficient effort and proximity to failure [143,144]. However, this broader literature cannot be directly equated with ribosome-biogenesis outcomes. Human studies specifically comparing different loading zones while measuring pre-rRNA, total RNA, or mature rRNA remain scarce.

The most defensible interpretation is that load intensity probably matters indirectly through its effects on mechanical tension, motor-unit recruitment, and the feasibility of accumulating productive volume [45,145]. Heavier loads may create a different force profile and require fewer repetitions to recruit high-threshold motor units, whereas lighter loads significantly increase local metabolic strain and prolong time under tension [145]. Yet the presence of these distinct physiological stressors does not establish that one loading zone is superior for nucleolar expansion. Until human studies directly compare such conditions using ribosomal endpoints, intensity of load should be presented as an important contextual variable rather than as an independently confirmed regulator of translational capacity.

#### 6.1.3. Effort, Proximity to Failure, and Fatigue Within the Set

Effort level is likely to be more relevant than absolute load when discussing the stimulation of hypertrophic adaptation. Meta-analytic evidence indicates that training to momentary failure is not clearly superior to non-failure training for hypertrophy when sets are performed with high effort (e.g., 1–3 repetitions in reserve) and total work is reasonably controlled [146]. In the context of ribosome biogenesis, this finding is important because it challenges the assumption that maximal within-set fatigue necessarily produces a superior ribosomal stimulus. At present, no definitive human trials show that training to absolute failure leads to greater pre-rRNA induction or larger total RNA accretion than training with a slight buffer.

Approaching failure may be useful insofar as it helps ensure full motor-unit recruitment and sufficient mechanotransduction [147]. However, pushing every set to absolute failure exponentially increases neuromuscular fatigue, prolongs recovery demands, and reduces the quality of subsequent sets or sessions [148]. Since ribosome biogenesis depends on the repeated accumulation of a productive stimulus across time, a strategy that consistently damages overall training quality could prove counterproductive. A related issue is the use of velocity loss thresholds as an operational way to monitor within-set fatigue. Reviews of this literature suggest that greater velocity-loss thresholds (e.g., >40%) drastically increase metabolic disruption and fatigue, but their superiority for hypertrophy relative to more moderate thresholds (e.g., 15–20%) is inconsistent and often inferior [149,150]. Thus, velocity loss can reasonably be framed as a useful fatigue-management concept to preserve the anabolic environment, but not yet as a validated determinant of translational-capacity expansion.

#### 6.1.4. Training Frequency and Distribution of the Weekly Stimulus

When weekly volume is equated, training frequency does not appear to exert a strong independent effect on hypertrophy in the general literature [151]. Even so, frequency remains conceptually relevant in a ribosome-biogenesis framework because it may influence the timing and repetition of the intracellular signals that drive pre-rRNA transcription. A given weekly volume distributed across more sessions may create more frequent, discrete transcriptional “pulses” of Pol I activation, potentially reducing the need for very high within-session fatigue [152]. Conversely, concentrating the same volume into fewer sessions may increase local exhaustion and compromise stimulus quality. At present, however, these ideas remain mechanistically plausible rather than experimentally established. There are no adequately controlled human trials directly comparing low- and high-frequency resistance training with matched weekly volume and repeated ribosomal measurements. Accordingly, frequency should be discussed as a variable that may shape the distribution of ribosome-biogenesis signaling opportunities, but not as one with a clearly demonstrated independent effect.

#### 6.1.5. Exercise Modality and Mode of Contraction

Exercise selection and modality also deserve consideration. Multi-joint and single-joint exercises, machine-based and free-weight movements all produce different profiles of force application, stabilization requirements, and central fatigue. However, the general literature demonstrates that when effort is matched, these modalities promote similar localized hypertrophic adaptations [153,154]. The ribosomal literature provides almost no direct basis for claiming that one of these broad categories is inherently superior for stimulating ribosome biogenesis [45]. The most realistic applied interpretation is that the selected exercises should allow the target musculature to be safely exposed to sufficient mechanical tension while preserving the ability to accumulate productive weekly volume. The same general logic applies to contraction characteristics (eccentric vs. concentric emphasis). Although these variables are debated in the general hypertrophy literature [143,144], the number of human studies measuring ribosomal endpoints under such comparisons remains too small to justify a hierarchy of superiority.

#### 6.1.6. Blood-Flow Restriction Training

One training-related strategy that does have direct relevance is blood-flow restriction (BFR) training. In a six-week study by Sieljacks et al., low-load BFR exercise and traditional high-load resistance exercise produced similar increases in cumulative myofibrillar protein synthesis and ribosomal biogenesis in healthy males [155]. This is one of the most important practical findings in the field because it demonstrates that high external loads are not necessary to stimulate ribosome-related adaptation in healthy individuals, likely due to the compensatory increase in cellular swelling and localized metabolic stress [156]. Although BFR is frequently utilized in rehabilitation, during periods of reduced joint tolerance, or when heavy loading is otherwise impractical [156], it must be explicitly noted that practical recommendations for clinical or sarcopenic populations are currently based on limited ribosomal-specific evidence. While BFR may provide a theoretically viable alternative for preserving or stimulating translational-capacity-related processes in these cohorts [155,156], its effectiveness depends on appropriate cuff pressure and individual tolerance, meaning it should be implemented cautiously and not generalized indiscriminately. Mechanistically, low-load BFR exercise has been shown to activate mTORC1/p70S6K1 signalling to a degree comparable with high-load resistance training [155,156] and, in parallel, to elevate Pol I-dependent transcription markers and ribosomal RNA species [155], providing a coherent low-load route to ribosomal biogenesis that combines partial mTORC1 activation with sufficient metabolic stress to drive nucleolar output.

#### 6.1.7. Continuity of Training, Interruptions, and Concurrent Training

Training continuity appears to be relevant to the maintenance of ribosomal adaptation. Hammarström et al. showed that the increase in total RNA observed during the early training phase was rapidly reduced after short-term cessation, indicating that ribosome content is not only trainable but also at least partly reversible [141]. This finding is practically important because it suggests that interruptions in training may compromise the translational apparatus that had begun to develop. While carefully managed reductions in training stress (deloads) may be useful to dissipate fatigue [142], complete interruption risks erosion of the ribosomal pool [141]. Furthermore, as established in previous sections, concurrent training, where endurance exercise is performed in close temporal proximity to resistance training, can profoundly attenuate elements of the ribosomal signaling response compared with resistance training alone [157]. Endurance volume, timing, and modality should be managed carefully to reduce interference with the translational-capacity response.

### 6.2. Nutrition

#### 6.2.1. Total Daily Protein Intake: Requirement Versus Optimization

Because amino acid availability and protein supplementation are well-established modulators of muscle protein synthesis and resistance-training adaptations [19,51], it may be biologically tempting to extrapolate that higher total daily protein intake should also directly expand the ribosomal pool. This inference is understandable, because ribosome assembly requires the synthesis and nucleolar import of ribosomal proteins, together with the coordinated activity of RNA polymerases I, II, and III [5,23,56]. However, this does not mean that protein-induced changes in muscle protein synthesis, fat-free mass, or strength can be interpreted as direct evidence of increased rDNA transcription or ribosome accumulation. These are related but non-equivalent biological endpoints.

Nutritional interpretation should therefore begin by distinguishing physiological requirement from performance-oriented optimization. For healthy adults, the European Food Safety Authority established a Population Reference Intake of 0.83 g/kg/day, and classic meta-analyses reached a similar order of magnitude using nitrogen-balance methodology [158,159]. In resistance-training contexts, protein supplementation has been examined as a strategy to improve lean-mass and strength outcomes [51]. However, regardless of how the magnitude or generalizability of those effects is interpreted, such outcomes should not be conflated with direct ribosomal endpoints. Evidence for changes in fat-free mass, strength, or acute MPS does not, by itself, demonstrate increased 45S pre-rRNA transcription, mature rRNA accumulation, total RNA content, or RNA:DNA ratio.

This distinction is especially important because the direct human evidence linking total daily protein intake to ribosome biogenesis remains limited. By contrast, the ribosomal-specific literature reviewed above more directly implicates repeated mechanical loading, recoverable training volume, training continuity, blood-flow restriction under appropriate conditions, and the timing of concurrent endurance exercise as practical modulators of ribosome-related outcomes [35,141,155,157]. Thus, the most defensible interpretation is that adequate protein availability is permissive for hypertrophic adaptation: it provides amino-acid substrate for muscle protein synthesis and ribosomal-protein production, but higher total protein intake has not been independently established as a direct driver of rDNA transcription or ribosomal expansion in human skeletal muscle.

#### 6.2.2. Protein Distribution and Leucine-Rich Feeding

Protein distribution is another nutritional variable with plausible relevance. A more even distribution of protein intake across meals increased 24-h muscle protein synthesis in healthy adults compared with a skewed pattern, and post-exercise protein ingestion stimulates synthesis in a dose-dependent manner up to a moderate per-meal level [160,161]. These findings suggest that the temporal pattern of amino acid availability can influence the integrated anabolic environment. Even so, increased 24-h MPS does not automatically establish greater ribosome biogenesis.

Similarly, leucine-rich feedings can amplify the stimulation of MPS, particularly in older adults or in contexts of anabolic resistance [111,162]. However, strong evidence for leucine as an acute regulator of translational *efficiency* (via mTORC1) does not amount to strong evidence for leucine as a direct stimulator of long-term ribosome *accretion*. The most accurate interpretation is that leucine-rich protein sources may help optimize the signaling environment surrounding repeated feeding and training opportunities, but their specific long-term effect on translational capacity remains underexplored.

#### 6.2.3. Energy and Carbohydrate Availability

Energy availability may be even more important than total protein intake in determining whether ribosome biogenesis can be supported over time. Ribosome production is metabolically expensive, and there is good physiological reason to expect it to be constrained under caloric deficit [163]. Human work has shown that acute energy deprivation reduces MPS and alters intracellular anabolic signaling [164]. Although protein ingestion and resistance exercise can partly mitigate these negative effects, severe or chronic energy deficits are highly likely to impair the expansion of translational capacity [165]. In practical terms, energy adequacy likely determines whether the body can afford the biosynthetic cost of nucleolar expansion.

Carbohydrate availability should be framed more conservatively. Sufficient carbohydrate intake may support training quality, glycogen restoration, and the ability to sustain productive effort across multiple sessions. However, direct evidence that carbohydrate feeding itself enhances ribosome biogenesis is weak. Lian et al. demonstrated that glucose ingestion before and after resistance-training sessions did not augment ribosome biogenesis in moderately trained young adults [52]. Carbohydrates should not be portrayed as direct stimulators of translational capacity; their value is indirect, preserving performance quality to support the training environment necessary for ribosome accumulation.

### 6.3. Supplementation and Other Modulatory Factors

#### 6.3.1. Creatine as an Indirect Energetic Support for Ribosome Biogenesis

At present, no nutritional or supplemental intervention discussed in this section has been shown to directly drive ribosome biogenesis in human skeletal muscle. Protein intake above the minimum required to sustain muscle protein synthesis is permissive of biogenesis but is not, by itself, a driver of rDNA transcription [51] (see Section 6.2.1). Creatine [166,167], citrulline [168,169], and leucine-rich feedings [111,162] are best interpreted as compounds that help maintain a permissive metabolic and signalling environment in which mechanical loading can drive nucleolar expansion. Where Table 3 and Table 4 list a level of direct ribosomal support as low or very low, this rating reflects the absence of direct human ribosomal evidence rather than an absence of mechanistic plausibility. High-confidence direct effects are restricted to the mechanical-load-dependent training variables (volume, proximity to failure, blood-flow restriction) for which human ribosomal-end measurements are available [35,145,155].

Among nutritional supplements, creatine has one of the strongest evidence bases for supporting resistance-training adaptations. Meta-analytic evidence indicates that creatine supplementation combined with resistance training may provide a small additional benefit for directly measured muscle hypertrophy [166]. Its relevance to ribosome biogenesis, however, should be framed as mechanistically plausible rather than directly demonstrated.

As outlined in earlier sections, high-volume resistance exercise imposes substantial energetic stress on the myofiber, increasing the likelihood of AMPK activation when ATP turnover outpaces resynthesis [60]. Because AMPK can restrain anabolic processes, including ribosomal RNA synthesis, any intervention that improves intracellular energy transfer may help preserve a more permissive environment for nucleolar activity [60]. In this context, creatine supplementation expands intramuscular phosphocreatine availability and may enhance the creatine kinase/phosphocreatine shuttle, which facilitates the spatial and temporal transfer of high-energy phosphate between mitochondria and sites of ATP consumption [167].

Rather than directly stimulating the nucleolus, creatine may therefore help buffer local energetic strain during repeated contractions, slow the rise in AMP- and ADP-related energetic stress, and reduce the likelihood of an exaggerated AMPK response. Nevertheless, direct human evidence that creatine supplementation increases 45S pre-rRNA expression, total RNA accretion, or RNA Polymerase I activity in skeletal muscle is currently lacking. Accordingly, creatine is best interpreted as a plausible indirect supporter of ribosome biogenesis, not as a proven direct activator of nucleolar output [166,167].

#### 6.3.2. Citrulline

Citrulline has attracted interest because human studies suggest it can stimulate MPS in specific nutritional contexts, raising the possibility that it may influence how energy and substrates are partitioned toward protein synthesis [168,169]. This makes citrulline biologically relevant within the broader discussion of anabolic support, particularly because any intervention that helps sustain net protein synthetic conditions could, in theory, favor the environment in which translational capacity is expanded over time. However, the current human literature linking citrulline directly to ribosomal endpoints such as total RNA accretion, 45S pre-rRNA expression, or sustained nucleolar activation remains very limited. Accordingly, citrulline should be framed as a compound with mechanistic plausibility and potential indirect relevance, but with unestablished modulatory power in relation to ribosome biogenesis.

#### 6.3.3. Thermal Strategies and Hypoxia

Thermal strategies show divergent evidence. Heat exposure remains speculative, with inconsistent evidence regarding its ability to enhance ribosome biogenesis [170]. Conversely, cold-water immersion has clearer mechanistic evidence, but in an unfavorable direction for hypertrophy phases. Figueiredo et al. showed that post-exercise cold-water immersion attenuated markers associated with ribosome biogenesis compared with active recovery [88]. This indicates that repeated immediate post-training cold exposure may interfere with the molecular environment favorable to translational-capacity expansion [171]. Finally, acute normobaric hypoxia has been shown to attenuate resistance-exercise-induced phosphorylation of ribosome-related signaling proteins (UBF and TIF-1A), though its chronic implications for translational capacity remain unresolved [172].

It is also worth acknowledging the pharmacological literature: anabolic–androgenic steroids consistently produce among the largest documented increases in human muscle protein synthesis, and the available molecular evidence indicates that these increases are supported not only by elevated translational efficiency (mTORC1/p70S6K1 signalling) but also by an expansion of ribosomal capacity, including upregulation of total RNA, ribosomal protein expression, c-Myc, and satellite-cell-mediated myonuclear addition. Although these compounds fall outside the scope of legitimate training and clinical practice, their effects on the nucleolar machinery reinforce the central thesis of this review—that any intervention capable of producing meaningful long-term hypertrophy ultimately converges on expanding translational capacity, not only on transiently amplifying translational efficiency.

### 6.4. Synthesis of Practical Applications

To synthesize the practical application of these variables, the current evidence supports a clear hierarchy of confidence. The strongest direct human support for modulating ribosome biogenesis lies with the organization and continuity of resistance training, especially volume-related exposure, and with blood-flow restriction when high loading is not feasible. Nutritional optimization and supplementation primarily act in a permissive or indirect role, safeguarding the energetic and metabolic environment required for the nucleolus to function optimally. These practical implications and the strength of their underlying evidence are summarized in Table 3 and Table 4.

## 7. Emerging Molecular Mechanisms Underlying Ribosomal Bottleneck

The current literature on skeletal muscle hypertrophy has consistently identified total RNA accretion and RNA Polymerase I activation as central processes associated with increases in muscle size [33,78]. However, interpreting ribosome biogenesis solely as an expansion in the number of equivalent translational units provides only a partial view of nucleolar biology. As methodological approaches in human muscle research increasingly incorporate single-nucleus sequencing and spatial transcriptomics [80,81], greater attention is being directed toward the regulatory mechanisms that shape nucleolar function, composition, and adaptive flexibility. In this context, a key challenge for the field will be to determine how skeletal muscle fibers modulate nucleolar activity to sustain large and persistent phenotypic adaptations [173].

From this perspective, future work should move beyond the quantification of ribosomal content and examine the molecular processes that regulate nucleolar output. These include chromatin remodeling at rDNA loci, the possibility of ribosomal heterogeneity, the contribution of non-coding RNAs, the role of nuclear mechanotransduction, and the systems involved in ribosomal quality control and turnover [174,175]. A more detailed understanding of these regulatory layers may refine current models of muscle adaptation and could eventually inform both clinical strategies targeting muscle loss and exercise programming in high-performance settings.

### 7.1. rDNA Accessibility and Epigenetic Regulation

Nucleolar activity is closely linked to the epigenetic configuration of ribosomal DNA (rDNA) loci [121]. Under basal conditions, a substantial proportion of the multiple rDNA repeats located on acrocentric chromosomes remain transcriptionally inactive, being associated with condensed chromatin and higher levels of DNA methylation [176]. This chromatin state constrains access of the transcriptional machinery to the rDNA promoter, thereby limiting RNA Polymerase I recruitment regardless of the presence of upstream anabolic signaling [177]. Accordingly, increased translational capacity depends not only on signaling activation, but also on the permissive structural state of the rDNA template.

Mechanical loading appears to influence this process through chromatin remodeling pathways. Foundational cellular models demonstrate that acetylation-mediated remodeling of the nucleolus is a primary driver of transcriptional activation [178]. In skeletal muscle, resistance exercise is thought to mirror this by promoting the recruitment of histone acetyltransferases (HATs) to nucleolar regions. Acetylation of lysine residues on histone tails reduces the affinity between histones and DNA, contributing to a more open chromatin configuration that facilitates transcriptional access to rDNA [178,179]. In parallel, repressive complexes such as the nucleosome remodeling and deacetylase (NuRD) complex are biologically known to establish and maintain the poised or silenced state of rRNA genes [180]. The subsequent suppression of these repressive complexes following mechanical overload reduces the maintenance of this silenced rDNA state, thereby allowing for robust nucleolar expansion.

DNA methylation of the rDNA promoter represents an additional level of regulation with possible implications for longer-term adaptations. DNA methyltransferases (DNMTs) can methylate CpG sites within promoter regions and thereby reduce binding of upstream binding factor (UBF), a key component of Pol I transcriptional initiation [181]. Human studies examining resistance training adaptations suggest that repeated loading may be associated with reduced methylation at rDNA loci and with lower expression of specific DNMTs [121,182]. This mechanism has been discussed in relation to the concept of muscle memory, insofar as a more permissive epigenetic profile could facilitate renewed transcriptional activation during retraining [124,182].

Additional nucleolar regulators may also contribute to this epigenetic plasticity. Among them, SIRT7 has attracted interest because of its prominent nucleolar localization and its involvement in the regulation of Pol I-dependent transcription [183]. In contrast to the broadly repressive actions often attributed to other sirtuins, SIRT7 appears necessary for preserving active rDNA transcription under conditions of cellular growth and stress [183,184]. Given its dependence on intracellular NAD+ availability, SIRT7 may provide a mechanistic link between cellular energetic status and nucleolar function [184].

Taken together, these observations suggest that rDNA accessibility is an important determinant of ribosome biogenesis in skeletal muscle. Future studies in humans should seek to characterize the time course and magnitude of these epigenetic responses to different loading paradigms. Clarifying the mechanical conditions most strongly associated with chromatin opening at rDNA loci could have practical implications for training design and may help explain part of the inter-individual variability in hypertrophic responsiveness [177,181].

### 7.2. Ribosomal Heterogeneity and Selective Translation

A major conceptual development in molecular biology has been the re-evaluation of the ribosome as a potentially heterogeneous rather than fully invariant structure. Historically, the mammalian 80S ribosome was viewed as a uniform translational machine with equivalent functional properties across contexts [185]. More recent work, however, has proposed that variation in ribosomal protein composition and rRNA modification patterns may generate ribosome populations with distinct translational preferences [186,187]. This framework, often referred to as ribosomal heterogeneity or specialized ribosomes, has opened new lines of inquiry into the relationship between ribosome composition and phenotypic adaptation.

**Figure 7:**
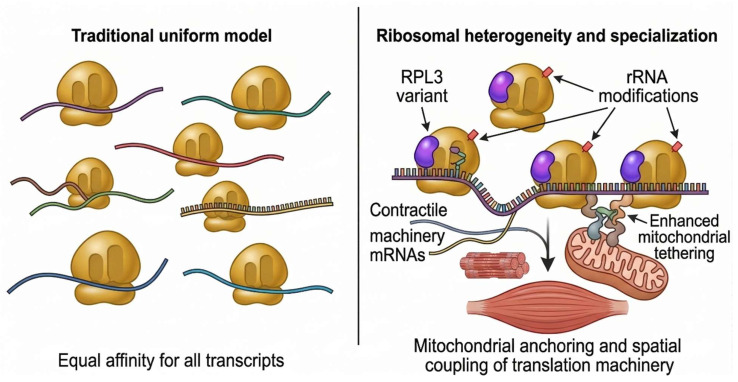
Ribosomal heterogeneity and spatial specialization in skeletal muscle adaptation. This conceptual schematic illustrates the shift from the traditional view of ribosomes as uniform machines to the emerging paradigm of ribosomal heterogeneity. The left panel shows standard ribosomes with an identical composition (predominantly containing the striated muscle-specific RPL3L paralog) translating different mRNAs in the sarcoplasm. The right panel depicts a mechanically overloaded muscle fiber in which nucleolar activity has altered ribosomal composition. Newly synthesized ribosomes incorporate the ubiquitous RPL3 paralog instead of RPL3L. According to this model, these customized ribosomes do not necessarily exhibit altered transcript selectivity, but rather display enhanced affinity for mitochondrial tethering. This spatial specialization physically couples the active translational machinery to the mitochondria, maximizing the local ATP supply required to sustain the massive energetic cost of synthesizing contractile machinery. This proposed structural specialization represents a potential additional layer of translational optimization. Beyond supplying ATP, mitochondrial tethering may also serve as a signalling interface: local mitochondrial-derived metabolites such as acetyl-CoA, α-ketoglutarate, S-adenosyl-methionine, and NAD^+^/NADH ratios, together with mitochondrial Ca^2+^ and redox flux, can influence rDNA transcription, ribosomal RNA modification, and nucleolar architecture, suggesting that mitochondria-ribosome coupling extends well beyond local energy supply into metabolic regulation of translational capacity.

In skeletal muscle, such a model may be relevant for understanding how translational machinery supports specific growth programs. Hypertrophy does not simply require increased protein synthesis in a generic sense; it depends on the sustained production of large structural and contractile proteins, including myosin heavy chain and titin [188]. While early hypotheses proposed that mechanical overload might favor the assembly of ribosomes specifically tailored to translate these structural mRNAs, the best-documented evidence of ribosomal specialization in skeletal muscle points toward a spatial and energetic adaptation (Figure 7). This is characterized by the striated muscle-specific paralog RPL3L, which is prevalent in resting tissue. Following hypertrophic overload, RPL3L expression plummets and is replaced by the ubiquitous isoform RPL3. Rather than altering mRNA selectivity for contractile proteins, this paralog switch enables the RPL3-containing ribosomes to physically anchor to mitochondria, maximizing the local ATP supply required to sustain the massive energetic cost of hypertrophy [187,189].

This possibility is further supported by the extensive post-transcriptional processing undergone by the 45S pre-rRNA. Before maturation, this precursor transcript is subject to more than 200 site-specific modifications, predominantly 2′-O-methylation and pseudouridylation [190]. These modifications contribute to ribosomal structure and function, particularly within domains relevant to decoding and translational fidelity [191]. Recent data suggest that the pattern of rRNA modification may change during periods of cellular growth, including modifications catalyzed by complexes such as dyskerin (DKC1) [190,192]. In experimental models, disruption of specific pseudouridylation events impairs translation of structurally demanding mRNAs, which may be especially relevant for the synthesis of major contractile proteins [192].

The implications for exercise physiology are potentially substantial. If ribosome composition influences which transcripts are preferentially translated, then the adaptive outcome may depend not only on the quantity of ribosomes produced but also on their molecular characteristics. This could offer one explanation for the interference effect observed during concurrent training: rather than entirely suppressing ribosome biogenesis, endurance-oriented stimuli may bias translational machinery toward proteins involved in oxidative metabolism and mitochondrial adaptation, whereas resistance exercise may favor ribosomal configurations more suitable for myofibrillar accretion [188,193].

To assess these possibilities, future studies will need to complement bulk RNA measurements with approaches capable of resolving ribosome composition and function more directly. Single-ribosome proteomics, ribosome profiling, and cryo-electron microscopy applied to human muscle tissue may prove especially informative [185,189]. Such work could substantially refine current interpretations of translational capacity in hypertrophying muscle.

### 7.3. Non-Coding RNAs and Nucleolar Regulation

The regulation of ribosome biogenesis cannot be fully understood by focusing exclusively on protein-coding genes. Increasing evidence indicates that several classes of non-coding RNAs, including long non-coding RNAs (lncRNAs), microRNAs (miRNAs), and small nucleolar RNAs (snoRNAs), participate in the control of nucleolar activity and ribosome production [194,195]. These molecules appear to influence transcriptional regulation, RNA processing, and translational assembly at multiple levels.

Among lncRNAs, several candidates have been implicated in skeletal muscle growth and nuclear regulation. However, it is crucial to explicitly note that current evidence largely stems from in vitro cell culture and animal models of myogenesis rather than adult human resistance training. For instance, recent discoveries in murine models reveal that some transcripts originally classified as putative lncRNAs encode hidden micropeptides that critically regulate muscle performance and intracellular signaling [196]. Meanwhile, specific lncRNAs (such as Myoparr) act as crucial promoters of cellular growth and myogenic activity, physically interacting with transcription factors to drive adaptation primarily during in vitro myogenic differentiation [195]. More broadly, extrapolating these basic developmental models to exercise physiology suggests that if the mechanical stimulus fails to orchestrate this intricate non-coding network, the entire adaptive and factory expansion process remains architecturally stalled [197]. Nevertheless, translating these specific lncRNA mechanisms to in vivo human hypertrophy requires further validation.

MicroRNAs provide another layer of post-transcriptional regulation. Because ribosome assembly depends on the coordinated production of ribosomal proteins in the cytoplasm, miRNAs capable of repressing transcripts encoding these proteins could indirectly constrain ribosome biogenesis [194]. Muscle-enriched miRNAs such as miR-1 and miR-133a have been implicated in the control of growth-related pathways and may modulate the translation of genes involved in ribosomal assembly or myogenic adaptation [198,199]. While the functional targets of these miRNAs have been extensively characterized in developmental biology and animal overload models, human in vivo trials confirm the downregulation of such miRNAs following mechanical loading. This mechanically induced suppression may therefore facilitate a more permissive environment for ribosomal protein synthesis and nucleolar output in adult human muscle, successfully bridging the gap between basic cell biology and applied exercise physiology.

Small nucleolar RNAs are of relevance because they directly participate in pre-rRNA processing and modification. As discussed above, snoRNAs guide site-specific cleavage and chemical modification of the 45S pre-rRNA, thereby contributing to proper ribosomal subunit maturation [190]. Furthermore, the functional diversity of snoRNAs, including members of the SNORD family, demonstrates their essential role in cellular adaptation and stress responses across various fundamental biological models [200]. Consequently, while direct human transcriptomic data tracing snoRNA dynamics during resistance training remain limited, the successful expansion of ribosome biogenesis in skeletal muscle likely requires coordinated adaptation not only at the level of transcription but also at the level of RNA processing and maturation guided by these small RNAs [200,201].

There is also growing interest in the possibility that non-coding RNAs contribute to intercellular communication within muscle tissue. Extracellular vesicles released by myofibers or satellite cells can transport miRNAs and other regulatory RNA species to neighboring cells in controlled experimental settings [202]. Although this area remains preliminary and largely restricted to in vitro or animal models, such mechanisms may participate in the coordination of adaptive responses across multinucleated fibers or between muscle-resident cell populations.

Overall, non-coding RNAs should be considered integral components of the regulatory network governing nucleolar function. Further clarification of their role in human exercise-induced adaptation may help explain why individuals differ in their translational response to mechanical loading and could reveal additional molecular targets relevant to muscle growth and preservation.

### 7.4. Nuclear Mechanotransduction and the YAP/TAZ Pathway

Conventional models of skeletal muscle hypertrophy have largely emphasized biochemical signaling cascades initiated at the sarcolemma, particularly those involving IGF-1, Akt, and mTORC1 [16,18,53]. Although these pathways are clearly relevant, they do not fully explain how high mechanical tension can directly influence transcriptional programs linked to growth [144,203]. Increasing attention has therefore been directed toward nuclear mechanotransduction, whereby physical forces acting on the myonucleus influence gene expression and nucleolar activity, in part through YAP/TAZ signaling [204,205].

The myonucleus is mechanically connected to the cytoskeleton and extracellular matrix through the LINC (linker of nucleoskeleton and cytoskeleton) complex [41,110]. As a result, external mechanical loading can be transmitted to the nucleus, altering its shape and mechanical state. Experimental studies indicate that nuclear deformation can affect pore dynamics, chromatin organization, and the intranuclear trafficking of mechanosensitive regulators [204,205,206]. These events provide a direct route through which loading conditions may modulate transcription independently of classical endocrine signals.

YAP and TAZ are among the best-characterized mediators of this process. Under low mechanical stress, these cofactors are typically retained in the cytoplasm in a phosphorylated state [203,204]. In response to increased mechanical loading, they translocate to the nucleus and interact with TEAD transcription factors, thereby promoting the expression of genes involved in cell growth, cytoskeletal remodeling, and ribosome biogenesis [203,206,207]. Evidence from cellular and animal models suggests that this pathway may contribute to nucleolar expansion and translational adaptation during muscle growth [204,207].

This framework may also be relevant to the attenuation of hypertrophic responses over time. As fibers enlarge and cytoskeletal organization changes, the transmission of force to the myonucleus may be altered. Increased intracellular stiffness could reduce nuclear deformation under a given external load, thereby attenuating mechanosensitive transcriptional signaling even if training volume remains substantial [110,206]. Such a mechanism may contribute to plateaus in adaptation and may partially explain why variation in exercise execution, range of motion, or loading profile can influence hypertrophic outcomes [203,207].

Further work in humans will be required to determine the relative contribution of nuclear deformation, stretch, and intracellular force transmission to YAP/TAZ activation in vivo. Advanced imaging and biomechanical approaches may be particularly useful in this regard [110]. A better understanding of these processes would support a more integrated view of resistance training as both a biochemical and a biomechanical stimulus.

### 7.5. Ribosomal Turnover and Quality Control

An expanded translational capacity is unlikely to be advantageous unless ribosomal integrity is also maintained. Ribosome biogenesis therefore needs to be considered alongside the systems responsible for monitoring ribosomal function, removing damaged components, and preserving translational fidelity [64,208]. This issue may be especially relevant in skeletal muscle, where repeated contractile activity, oxidative stress, and fluctuations in energy availability can challenge ribosome stability [209,210].

One of the main protective systems involved is ribosome-associated quality control (RQC). When translation stalls due to damaged mRNA, amino acid insufficiency, or structural ribosomal defects, ribosome collisions can occur along the transcript. These events are detected by surveillance mechanisms that include the E3 ubiquitin ligase ZNF598, which helps mark defective translational complexes for resolution and degradation [208,209]. By limiting the persistence of stalled ribosomes, RQC contributes to proteome quality and reduces the accumulation of aberrant translation products [208,211].

Defective ribosomal components may subsequently be removed through ribophagy, a selective form of autophagy targeting ribosomes [64]. NUFIP1 has been identified as one of the receptors involved in this process, particularly in conditions requiring ribosomal recycling [212]. Although excessive activation of ribophagy could compromise anabolic potential, a basal level of selective turnover is likely necessary to maintain a functional ribosomal pool during repeated cycles of loading and recovery [211,212].

This balance between synthesis and removal suggests that translational adaptation has an important qualitative dimension. If anabolic signaling drives high rates of translation without sufficient recovery or quality control, the resulting increase in translational errors may activate stress responses that ultimately impair protein synthesis efficiency [208,209,210]. From an applied perspective, this reinforces the notion that recovery periods are relevant not only for substrate restoration and tissue repair, but also for the maintenance of translational fidelity.

Future studies should aim to quantify ribosomal turnover in human skeletal muscle under different training and nutritional conditions. Dynamic proteomic approaches, including D_2_O labeling combined with mass spectrometry, may be particularly useful for examining how newly synthesized and degraded ribosomal pools are regulated in vivo [71,212]. Such work may help clarify how ribosome quality control contributes to long-term hypertrophic adaptation (Table 5).

## 8. Limitations and Future Directions

Despite the robust mechanistic models and empirical data presented in this review, the contemporary literature on ribosome biogenesis in applied sports science remains subject to important demographic and temporal limitations. Most in vivo human hypertrophy trials have been conducted in young, healthy, recreationally active males, leaving a substantial gap in our understanding of how these mechanisms operate across other populations. Data specifically examining nucleolar adaptation in female cohorts, particularly considering the potential modulatory effects of fluctuations in ovarian hormones on mechanotransduction, remain scarce [213,214,215]. In addition, most longitudinal hypertrophy interventions last only 6 to 12 weeks. Although this time frame captures the initial expansion of the ribosomal pool, it does not clarify longer-term plateaus in ribosomal accretion, rates of ribosomal decay, or the actual biological ceiling of translational capacity that may be reached by elite athletes over years of training [141,216].

Several methodological limitations of the present manuscript should also be acknowledged. Although a rigorous, systematic PRISMA-guided search strategy was used to establish the conceptual basis of this review, the final work is ultimately a narrative and integrative synthesis. As such, it remains inherently susceptible to selection bias and to the interpretive framework of the authors. More importantly, the marked heterogeneity in how primary studies quantify and normalize ribosomal RNA, from whole-muscle homogenates to single-fiber transcriptomics and RNA:DNA ratios, prevents the performance of a precise and methodologically sound quantitative meta-analysis [73]. Until the field adopts more standardized approaches for reporting absolute changes in translational capacity, comparisons across studies will remain largely inferential rather than strictly quantitative.

To address these methodological barriers, future research should move toward standardized and metabolically traceable protocols. In particular, the field should progressively move beyond static transcriptomic snapshots and adopt dynamic tracing techniques, especially oral deuterium oxide (D_2_O) administration combined with high-resolution mass spectrometry, to quantify the fractional synthetic rate of ribosomes in vivo. In parallel, biopsy timing should be more rigorously harmonized across laboratories. Capturing the acute transcriptional response (e.g., 45S pre-rRNA) requires sampling within a narrow 2- to 4-h post-exercise window, whereas assessment of mature ribosomal expansion requires longer-term follow-up across weeks. The combination of D_2_O tracing with emerging spatial transcriptomic approaches may help resolve not only whether ribosome biogenesis occurs, but also where it is localized within the multinucleated syncytium in response to different mechanical stimuli.

From an applied and clinical perspective, the next step will require long-term randomized controlled trials (>6 months) that manipulate specific training and nutritional variables while directly assessing nucleolar output. Well-controlled interventions examining the interaction between endurance and resistance training are needed to clarify how different volumes and intensities modulate the AMPK-Pol I axis over time. Likewise, understanding how specific nutritional conditions (e.g., severe energy restriction or particular amino acid profiles) affect ribosomal quality control and ribophagy pathways may have important clinical implications for sarcopenia and cachexia. Bridging the gap between the molecular biophysics of nuclear mechanotransduction and applied training periodization may ultimately refine evidence-based hypertrophy programming.

## 9. Conclusions and Practical Applications

### 9.1. Conclusions

The conceptual framework of skeletal muscle hypertrophy is undergoing an important shift. For decades, both researchers and practitioners have largely focused on the transient increases in translational efficiency regulated by the mTORC1 signaling cascade. However, the molecular evidence reviewed here suggests that acute signaling is primarily permissive. The main bottleneck for sustained macroscopic hypertrophy appears to be the structural expansion of the muscle fiber’s nucleolar machinery, namely an increase in translational capacity driven by RNA Polymerase I transcription and subsequent ribosome biogenesis.

This nucleolar capacity model helps explain the marked inter-individual heterogeneity observed in training responses, distinguishing high responders from individuals who show limited hypertrophic adaptation. It also offers a biologically plausible explanation for age-related anabolic resistance, in which the senescent nucleolus appears to become more rigid and less mechanosensitive. In addition, the integration of satellite cell fusion and epigenetic remodeling of rDNA promoters provides a strong molecular basis for the phenomenon commonly referred to as “muscle memory”. In this context, nucleolar expansion is not simply a transient metabolic condition, but rather a structural and epigenetic adaptation that may durably influence the growth trajectory of the fiber.

Ultimately, skeletal muscle hypertrophy can be understood as a problem of cellular infrastructure. Just as a manufacturing plant cannot sustainably increase output by relying only on faster signals delivered to a fixed number of workers, a muscle fiber cannot maintain large-scale protein accretion without expanding its translational machinery. Shifting the focus from transient signaling events toward the regulation of translational capacity may allow sports scientists and clinicians to design more precise and mechanistically grounded interventions to enhance adaptation.

### 9.2. Practical Applications

Based on the mechanistic and empirical evidence reviewed, practitioners, coaches, and clinicians should consider the following practical applications for optimizing hypertrophy:

- **Volume is the primary driver:** Sufficient and recoverable mechanical volume appears to be the most potent known stimulus for Pol I transcription. Therefore, to sustain the nucleolar stimulus throughout the microcycle, practitioners should prioritize the accumulation of an adequate weekly number of hard sets (e.g., ≥10 sets per muscle group). Importantly, this threshold is derived from general hypertrophy meta-analyses [100], rather than from studies specifically designed to determine a precise weekly dose–response relationship for ribosomal outcomes. Nonetheless, early-phase molecular evidence suggests that reaching this higher volume may help ensure that the anabolic signal exceeds the natural turnover of pre-existing ribosomes [35].
- **Manage the energetic bottleneck (interference effect):** Because ribosome biogenesis is highly ATP-demanding, severe energetic stress (e.g., high-intensity endurance training) activates AMPK, which can act as a brake on nucleolar activity. To maximize hypertrophy, concurrent endurance sessions should ideally be separated from resistance training by at least 6 to 24 h.
- **Distinguish protein optimization from necessity:** Although daily protein intakes of ~1.6 g/kg/day may help optimize the anabolic environment according to some studies, very high protein intakes alone do not appear sufficient to drive ribosome biogenesis in the absence of an adequate mechanical stimulus. In this sense, nutrition plays a permissive rather than primary role in nucleolar expansion.
- **Blood-flow restriction (BFR) is a viable alternative:** For populations that cannot tolerate high mechanical loads (e.g., individuals in rehabilitation or with severe sarcopenia), low-load BFR may provide a practical alternative capable of reaching the threshold needed to stimulate ribosome biogenesis, likely through local cellular swelling and metabolically mediated mechanotransduction.
- **Consistency outperforms extreme exhaustion:** Taking every set to absolute failure or using very high levels of velocity loss markedly increases neuromuscular fatigue and metabolic stress, which may compromise recovery of the nucleolar machinery. Leaving 1 to 3 repetitions in reserve may allow high-quality mechanotransduction while preserving the ability to accumulate productive weekly volume.
- **Energy deficits stall factory expansion:** Chronic or severe caloric restriction substantially limits the cell’s ability to support the metabolically costly process of ribosome assembly. Athletes aiming to maximize hypertrophy should therefore maintain sufficient energy availability to support expansion of translational capacity.

## Figures and Tables

**Table 1:** Methodological comparison of imaging and spatial techniques for resolving nucleolar dynamics in skeletal muscle.

Imaging Modality	Primary Target/Output	Spatial Resolution	Major Methodological Limitation
**Transmission electron microscopy (TEM)**	Ultrastructural nucleolar volume and density; DFC/GC compartmentalization	Sub-nanometer	Restricted to extremely small 2D fields of view; highly susceptible to sampling bias across the syncytium [30].
**Confocal immunofluorescence**	Localization of specific nucleolar proteins (e.g., fibrillarin, UBF, and c-Myc).	~200–300 nm	Insufficient resolution to fully visualize the precise epigenetic and internal architecture of the tripartite nucleolus [78].
**Super-resolution microscopy (STED/STORM)**	3D volumetric expansion of nucleolar compartments and rDNA accessibility.	~20–50 nm	High technological barrier; severe issues with skeletal muscle autofluorescence and deep-tissue penetration [54].
**Fluorescence in situ hybridization (FISH)**	Subcellular localization and absolute counts of 45S pre-rRNA and mature rRNA.	Cellular/Subcellular	Difficult to optimize probe hybridization in heavily cross-linked, protein-dense myofibrillar tissue [80].

**Table 2:** Phenotypic and molecular divergence between hypertrophic responders following resistance training [70,95,99].

Molecular/Cellular Variable	Extreme High Responders	Extreme Low Responders (Non-Responders)	Bottleneck Interpretation
**Acute mTORC1/p70S6K**	Robustly elevated	Equivalently or higher elevated	Efficiency signal is intact in both groups; not the limiting factor.
**45S pre-rRNA (Pol I)**	Sustained transcription	Blunted or highly transient	Failure to transmit mechanical signal to the nucleolus in non-responders.
**Total RNA accumulation**	Massive increase	Stagnant or negligible	High responders successfully widen the physical translational capacity.
**Myonuclear addition**	Significant SC fusion	Negligible SC fusion	Non-responders fail to expand the myonuclear domain (MND) architecture.
**RNA:DNA ratio**	Highly elevated	Unchanged	Confirming absolute factory expansion is the prerequisite for extreme growth.

**Table 3:** Practical implications of the main modulators of ribosome biogenesis.

Modulator	Practical Interpretation	Level of Direct Ribosomal Support
**Weekly training volume**	Primary training-related driver; likely beneficial when recoverable.	High
**Load intensity**	Important contextual variable, but no clear direct superiority established.	Low to moderate
**High effort/proximity to failure**	Likely necessary to ensure adequate stimulus; absolute failure not proven superior.	Low to moderate
**Training frequency**	May shape temporal distribution of ribosomal signaling; independent effect unclear.	Low
**Blood-flow restriction**	Viable low-load alternative when heavy loading is not feasible, though clinical ribosomal data are lacking	Low to moderate
**Training continuity**	Likely important for preserving ribosomal adaptation.	Moderate
**Concurrent training**	May attenuate ribosome-related signaling if poorly timed.	Moderate
**Total protein intake**	0.83 g/kg/day remains requirement benchmark; higher intakes reflect optimization, not necessity.	Low for direct outcomes
**Protein distribution**	Plausibly supportive via repeated anabolic opportunities.	Low
**Leucine-rich feeding**	Likely useful for acute anabolic responsiveness; direct ribosomal effect unclear.	Low
**Energy availability**	Likely important determinant of the feasibility of ribosome biogenesis.	Moderate indirect
**Carbohydrate availability**	Probably indirect support through training quality and recoverability.	Low
**Creatine**	Useful hypertrophy adjunct; energetic buffer to delay AMPK and protect Pol I.	Low (indirect)
**Citrulline**	Mechanistically interesting but insufficiently validated.	Very low
**Heat exposure**	Exploratory; no practical recommendation justified.	Very low
**Cold-water immersion**	May blunt a favorable ribosomal environment after hypertrophy training.	Moderate
**Hypoxia**	Mechanistically uncertain; no clear practical value established.	Very low

**Table 4:** Strength and directness of evidence for modulators of ribosome biogenesis.

Variable	Direct Human Ribosomal Evidence	Confidence of Practical Conclusion
Training		
**Volume**	Yes	High
**Intensity**	Limited	Low
**Effort/failure**	Limited	Low
**Frequency**	Indirect	Low
**Exercise modality**	Minimal	Very low
**Blood-flow restriction**	Yes (in healthy cohorts)	Low to moderate
**Continuity/cessation**	Yes	Moderate
**Concurrent training**	Yes	Moderate
**Nutrition**		
Total protein	Limited for ribosomal markers	Moderate for requirement, low for direct effect
Distribution	Indirect	Low
Leucine	Indirect	Low
Energy availability	Indirect to moderate	Moderate
Carbohydrate	Limited	Low
**Supplements and others**		
Creatine	Indirect	Low
Citrulline	Minimal	Very low
Heat	Minimal	Very low
Cold-water immersion	Yes	Moderate
Hypoxia	Limited	Very low

**Table 5:** Advanced molecular frontiers regulating the translational capacity bottleneck.

Frontier Category	Primary Target/Mechanism	Functional Role in Hypertrophy
**Epigenetic regulation**	rDNA promoter (HATs, SIRT7, DNMTs)	Physically uncoils heterochromatin to permit RNA Polymerase I access; establishes persistent “muscle memory” [178,183].
**Ribosomal heterogeneity**	Ribosomal protein composition and rRNA modification patterns	May influence translational selectivity and functional ribosome behavior during adaptation, although selective translation of hypertrophy-related transcripts in human skeletal muscle remains unproven [186,187].
**Non-coding RNAs**	lncRNAs, miRNAs, and snoRNAs	Contribute to the regulation of myogenic transcription, RNA stability, and pre-rRNA processing, thereby potentially influencing ribosome biogenesis at multiple levels [195,198].
**Nuclear mechanotransduction**	YAP/TAZ nuclear entry and TEAD-dependent transcription	Couples mechanical deformation to growth-related transcriptional programs upstream of c-Myc and ribosome biogenesis [204,205].
**Ribosomal quality control**	ZNF598 (collision sensing) & NUFIP1 (ribophagy)	Detects stalled translation and clears defective ribosomes, maintaining the high structural fidelity required for extreme accretion [208,212].

## Data Availability

No new data were created or analyzed in this study.
